# Re-Expression of Tafazzin Isoforms in TAZ-Deficient C6 Glioma Cells Restores Cardiolipin Composition but Not Proliferation Rate and Alterations in Gene Expression

**DOI:** 10.3389/fgene.2022.931017

**Published:** 2022-07-25

**Authors:** Gayatri Jagirdar, Matthias Elsner, Christian Scharf, Stefan Simm, Katrin Borucki, Daniela Peter, Michael Lalk, Karen Methling, Michael Linnebacher, Mathias Krohn, Carmen Wolke, Uwe Lendeckel

**Affiliations:** ^1^ Institute of Medical Biochemistry and Molecular Biology, University Medicine Greifswald, University of Greifswald, Greifswald, Germany; ^2^ Institute of Clinical Biochemistry, Hannover Medical School, Hannover, Germany; ^3^ Department of Otorhinolaryngology, Head, and Neck Surgery, University Medicine Greifswald, Greifswald, Germany; ^4^ Institute of Bioinformatics, University Medicine Greifswald, Greifswald, Germany; ^5^ Institute of Clinical Chemistry, Department of Pathobiochemistry, Medical Faculty, Otto-von-Guericke University Magdeburg, Magdeburg, Germany; ^6^ Institute of Biochemistry, University of Greifswald, Greifswald, Germany; ^7^ Department of General Surgery, Molecular Oncology, and Immunotherapy, Rostock University Medical Center, Rostock, Germany

**Keywords:** Barth syndrome, cardiolipin, cellular proliferation, gene expression, tafazzin, Barth syndrome (BTHS)

## Abstract

Tafazzin—an acyltransferase—is involved in cardiolipin (CL) remodeling. CL is associated with mitochondrial function, structure and more recently with cell proliferation. Various tafazzin isoforms exist in humans. The role of these isoforms in cardiolipin remodeling is unknown. Aim of this study was to investigate if specific isoforms like Δ5 can restore the wild type phenotype with respect to CL composition, cellular proliferation and gene expression profile. In addition, we aimed to determine the molecular mechanism by which tafazzin can modulate gene expression by applying promoter analysis and (Ingenuity Pathway Analyis) IPA to genes regulated by TAZ-deficiency. Expression of Δ5 and rat full length TAZ in C6-TAZ- cells could fully restore CL composition and—as proven for Δ5—this is naturally associated with restoration of mitochondrial respiration. A similar restoration of CL-composition could not be observed after re-expression of an enzymatically dead full-length rat TAZ (H69L; TAZMut). Re-expression of only rat full length TAZ could restore proliferation rate. Surprisingly, the Δ5 variant failed to restore wild-type proliferation. Further, as expected, re-expression of the TAZMut variant completely failed to reverse the gene expression changes, whereas re-expression of the TAZ-FL variant largely did so and the Δ5 variant to somewhat less extent. Very likely TAZ-deficiency provokes substantial long-lasting changes in cellular lipid metabolism which contribute to changes in proliferation and gene expression, and are not or only very slowly reversible.

## Introduction

The mitochondrial phospholipid cardiolipin (CL) has been linked to mitochondrial and cellular functions and to mitochondrial energy metabolism in particular. Accumulating evidence points to the existence of an association of the composition of CL with cellular proliferation (Schild et al., 2012; Murke et al., 2016; Ohlig et al., 2018; Gurtler et al., 2019). Although such a correlation could be demonstrated for several cell types, evidence for a causal relationship remains obscure.

CL composition shows a high degree of tissue and organ specificity (Schlame and Greenberg, 2017) and has been linked with a broad variety of cellular characteristics and, in particular, with mitochondrial structure and properties like mitochondrial membrane fluidity and cellular elasticity (Gurtler et al., 2019), oxidative phosphorylation (Raja and Greenberg, 2014), electron flow along respiratory chain complexes and electron leakage (Pfeiffer et al., 2003), regulation of the intrinsic pathway of apoptosis (McMillin and Dowhan, 2002), and mitochondrial fission and fusion (Frohman, 2015).

Recently, substantial alterations in CL composition could be forced by either supplementation of fatty acids or knock-out of the phospholipid remodeling enzyme, tafazzin (TAZ), respectively (Schild et al., 2020). Most notably, under the conditions applied, the effects on proliferation of C6 glioma cells appeared to be independent of CL composition (Gurtler et al., 2019).

The composition of molecular lipid species including CL is subject of change and constantly requires adaptation in response to physiological stimuli or pathophysiological conditions to meet metabolic and other cellular requirements (Kimura et al., 2016). The availability of fatty acids, whether free or bound in other cellular phospholipids, for CL *de novo* biosynthesis has been recognized as the most important factor for establishing CL composition in recent years (Oemer et al., 2020; Oemer et al., 2021; Oemer et al., 2022).

Tafazzin is an acyltransferase with key function in CL remodeling. Indeed, it is the most efficient remodeling system for CL (Ye et al., 2016). It mediates the exchange of fatty acids between cellular phospholipids and mono-lyso-phospholipids that may result from oxidative stress (Ye et al., 2016). However, it is not known if remodeling is the only function of tafazzin (Schlame, 2013). The tafazzin gene is mutated in patients with Barth syndrome, a genetic disorder associated with changes in CL composition and in the mono-lyso-CL/CL ratio, cardiomyopathy, skeletal muscle weakness, impaired mitochondrial structure and function, and neutropenia (Clarke et al., 2013). A tafazzin knock-out mouse model developed a heart and muscle phenotype similar to what is typically observed in Barth patients (Wang et al., 2020). Furthermore, knock-down and knock-out of tafazzin in rat C6 provoked similar changes in CL composition, mitochondrial respiration (oxidative phosphorylation, OXPHOS), mitochondrial morphology and ultrastructure, and is associated with decreased cell proliferation (Murke et al., 2016; Ohlig et al., 2018; Gurtler et al., 2019; Schild et al., 2020) as observed in Barth patients. Knock-down of tafazzin has been shown to affect cell cycle progression in neonatal ventricular fibroblasts (He et al., 2013). This mechanism very likely contributes to myopathy in Barth syndrome (Clarke et al., 2013). Recently, the overexpression or knock-down of TAZ, respectively, has been associated with alterations in proliferation of cervical cancer cells (Chen et al., 2017).

In addition to the full length human tafazzin gene comprising 11 exons four alternatively spliced transcripts variants are known to be expressed (Vaz et al., 2003). Until now, it is not established if these variants have redundant functionality in CL remodelling or contain additional differing functionalities. At least human full length (FL) tafazzin and the TAZ principle variant lacking exon 5 (Δ5) exhibit transacylase activity and, thus, have been shown to restore cardiolipin composition, mitochondrial respiration in tafazzin-deficient *D. melanogaster* (Xu et al., 2009).

Tafazzin-deficient C6 cells, C6-TAZ, have been extensively characterized before (Ohlig et al., 2018; Gürtler et al., 2019; Schild et al., 2020) with respect to CL composition, mitochondrial respiration, structure and membrane fluidity, and cell proliferation. Extending these findings, the results of this study additionally show massive changes in gene expression that occur in C6 cells in response to knock-out of tafazzin. Differential gene expression was verified and regulated genes were subjected to promoter and IPA analyses to identify possible underlying mechanisms. To what extent the different tafazzin isoforms are capable of reversing the effects of tafazzin knock-out in rat C6 glioma cells is not known and should be investigated in the present study. To this end, we (re-)expressed in tafazzin-deficient C6 cells the originally contained and deleted in C6 cells rat full length tafazzin, the human isoform lacking exon 5 (Δ5) and an enzymatically dead mutant (H69L) full length tafazzin and subsequently studied their impact on CL-composition, cell proliferation, and differential gene expression.

## Materials and Methods

### Cells

Rat C6 glioma cells (hereafter referred to as C6) were obtained from DSMZ (Braunschweig, Germany). Tafazzin (TAZ) was knocked out in C6 cells (C6 TAZ) using the CRISPR-Cas9 system as previously described (Gürtler et al., 2019).

### Stable (Re-)Expression of Tafazzin Variants in C6 and C6 TAZ Cells

Human tafazzin gene lacking exon 5 (Δ5) was kindly provided by Prof. Michael Schlame, NYU Medical center. The pCDNA 3.1 vector was linearized with restriction enzyme BglII (New England Biolab, United States) and the TAZ-gene containing fragment (6,217 bp) was used to transfect C6 and C6 TAZ cells using lipofectamine 2000 (Thermo Fisher Scientific, Waltham, United States).

cDNA encoding for rat wild-type tafazzin (accession #NM_001025748) and an enzymatically inactive variant of rat full-length tafazzin (H69L) were synthesized and cloned into XhoI/XbaI sites of the lentiviral transfer plasmid pLVX-IRES-Neo (Takara Bio Europe, Saint-Germain-en-Laye, France) as part of the gene synthesis service of BioCat (Heidelberg, Germany). Plasmid was cut using restriction enzymes NheI and EcoNI (New England Biolab, United States). Later, the fragment of plasmid with tafazzin gene (5,365 bp) was transfected in C6 and C6 TAZ cells using lipofectamine 2000. Stably transfected cells were selected with 1 mg/ml G 418 Geneticin (PAN Biotech, GmbH, Aidenbach, Germany) over a period of 14 days. All cells were maintained in DMEM medium supplemented with 3% fetal calf serum (FCS), 1% penicillin and streptomycin (cell culture medium) at 37°C with 5% CO_2_, and passaged every 2–3 days.

#### Cardiolipin Analysis

Extraction of lipids and CL analysis was performed as recently described (Ohlig et al., 2018; Gurtler et al., 2019). Similar to our previous study, we analysed (C18:2)3-CL as the representative of mono-lyso-CL (MLCL) and (C18:2)3-hydroxy-linolyl CL as indication for CL oxidation.

#### Extraction and HPLC-MS/MS Measurement of Oxylipins

Cell lysis and extraction of lipid mediators was performed as previously described (Schultz et al., 2020). In brief, internal standards were added and the cells were lysed using FastPrepTM lysing matrix D and a FastPrepTM homogenizer. Lysing cycle was repeated after a washing step. Combined supernatants were used for an alkaline hydrolysis followed by a solid-phase extraction.

Samples were measured with an Agilent^®^ HPLC system (1200), coupled to an Agilent^®^ 6460 Triple quadrupole mass spectrometer with a Jetstream ionization source using a dynamic multiple reaction monitoring (dMRM) method. Chromatographic and MS parameters are referred to in (Schultz et al., 2020). Data analysis was performed with Agilent Mass Hunter Qualitative and Quantitative Analysis software (version B.08.00 for both). For all detected oxylipins, a relative quantification was done by normalizing the area of the metabolite signal to the area of the signal of the internal standard compound (relative amount). 12-HETE-d8 was the internal standard for HETEs, EETs, HEPEs and HDHAs, and 13-HODE-d4 for HODEs, and 13-HOTrE. All relative amounts of the oxylipins were normalized to the cell number (10^6^ cells per sample).

#### Microarray Expression Analysis

Transcriptome analyses were performed using the Affymetrix GeneTitan Multi-Channel platform with Clarion S rat microarray and GeneChip WT Plus kit (Affymetrix Inc., Santa Clara, VA, United States). Expression profiling was done with pooled RNA from three independent biological replicates each of wild type C6 and tafazzin-deficient C6-TAZ cells. Total cellular RNA was isolated according to manufacturer’s protocol (Analytikjena RNA isolation kit, Jena, Germany) 1 µg RNA input was taken for gene expression analysis. Data were analysed as described (Bukowska et al., 2012). Significant differences in gene expression were identified using the following cut-off criteria: one-way analysis of variance with subsequent Benjamini and Hochberg false discovery rate multiple-testing correction on pair-wise comparisons, (ANOVA, *p* 0.05), signal correction statistics (ratio builder, *p* < 0.05), and fold-change 1.5-fold (for a complete list of significantly regulated genes, see Online Supplementary Table S1).

### Transcription Factor Binding Site Detection

For the TF binding site analysis we used the tool Ciiider (Gearing et al., 2019) to identify common TF binding sites in the detected up- and/or down-regulated genes from the Microarray expression analysis. Furthermore, we used the tool to search for common TF binding profiles in the specific Cholesterol pathway as well as for PPARG solely. Therefore, we downloaded the reference fasta and gtf files of *Rattus norvegicus* from the Ensembl database (Yates et al., 2020) and the JASPAR2020_CORE_vertebrates database (Castro-Mondragon et al., 2022) of TF binding profiles. For the analysis we used 1,500 nt cut-off for upstream and 500 nt cut-off for downstream sequences. The deficit cut-off was set to 0.15 for all analyses. To exclude unspecific TF binding profiles in the up- and downregulated gene lists we used a background gene list consisting of the non-affected genes in the microarray expression analysis (foldchange between 1 and +1). The performed enrichment analysis used a coverage *p*-value below 0.05 and a site *p*-value of 1.0 (for a complete list of significantly regulated genes, see Online Supplementary Table S1).

### In Silico-Pathway- and Functional Analysis of Microarray Data

In-silico-pathway- and functional analysis of differentially expressed genes was carried out using the commercial systems biology-oriented package Ingenuity Pathways Analysis (QIAGEN Inc., https://digitalinsights.qiagen.com/IPA) using the annotation details provided by Couture et al. (Couture et al., 2009) with their corresponding gene identifiers and expression values.

### Reverse Transcription Quantitative PCR

PCR Reverse transcription quantitative PCR (RT-qPCR) was applied to validate selected candidate genes that were identified as differently expressed in the microarray analyses C6 vs. C6-TAZ). Reactions were performed as previously described (Chilukoti et al., 2013; Chilukoti et al., 2015). Quantities of RPL13a mRNA were used to normalize cDNA contents. The specific primers applied are listed in Table 1.

**TABLE 1:** List of primers used in the study.

Gene	Upstream	Downstream
rPLP1	GCC CTG ACT GTT GTA TGG CT	AGG GAA ACT AGT GTG GCT GC
rCXCL1	CTG CAC CCA AAC CGA AGT CA	GAC GCC ATC GGT GCA ATC TA
rCPE	ACC TCC CTG TCG CAA GAA TG	CCA TCC TTA GCC GAG GTG AC
rDPP7	GGG GAG CAC ATC ACC TAG AC	GAA GGC TGC TAC TTA GGC CC
rPPARG	TCA AAG TAG AGC CTG CGT CC	TGG CAT TGT GAG ACA TCC CC
rCPM	CGA GGC AAG ATT GAC CCA GT	CAG CTC GTT TCC TTT CAC GC
rMVK	TCA TGG TGT GGT CGG AAC TG	GGT ACT TCG TGG GAC CTT GG
rENPEP	CCT CAC ATC CGG TGG TTG TC	TGG GTG ACG TTC TGC TTT CC
rSC5D	GAC CCT GGC AGC ACT GTA AT	GGT CGG CTT TCC TGG CTA AT
rHMGCR	TAG AGA TCG GAA CCG TGG GT	GCC CGT GTT TCA GTC CAG TA
rHSD17B7	CTG ACC AAA TAC TTG AGC GGC	TAG GAG GAG AGA TCA TCA TGG C
rCRLS1	GCA TTC ACT ACA GCT GCG TC	GCT GAA CAC CAA GAT CGG GA
rIL6	TCA TTC TGT CTC GAG CCC AC	GTC TCC TCT CCG GAC TTG TG
rILa	AGT GGA ACC AGC CCG ACA TA	TAT CCT ACC CAT CCG GCA CT
RPLP13a	CTG GTA CTT CCA CCC GAC CTC	GGA TCC CTC CAC CCT ATG ACA

### Western Blot

C6 (w/o Δ5 or FL or Mut) and C6 TAZ (w/o Δ5 or FL or Mut) cells were lysed in RIPA lysis buffer (50 mM Tris HCl pH 7.5, 5 mM EDTA, 100 mM NaCl, 0.5% Triton, 10% glycerol, 10 mM K_2_HPO4 0.5% NP-40) 0.5% pre-warmed DOC (deoxycholic acid), 1 mM NaVO_3_, 20 mM NaF, 0.1 mM PMSF, 20 mM glycerol-2-phosphate, 0.5% Tween 20, and 0.5% SDS) supplemented with a 1x protease-inhibitor cocktail (Roche Diagnostics GmbH, Mannheim, Germany) on ice for 45–60 min. After centrifugation for 30 min at 17,000 g at 4°C, the protein concentration in the lysates was determined by Bradford method. 40 μg of protein lysate was then separated by SDS-PAGE and transferred to a nitrocellulose membrane, followed by blocking with 1x non-protein solution, Roti (Carl Roth GmbH Karlsruhe, Germany) for 1 h, at RT. After washing three times with Tris-buffered saline with 0.1% Tween 20 (TBST), the membrane was incubated with primary mouse anti-tafazzin antibody (Santa Cruz Biotechnology, 1:1000 in TBST, 5% BSA, and 0.03% NaN3) at 4°C overnight, followed by secondary anti-mouse antibody conjugated with horseradish peroxidase (Cell Signaling technology, 1: 10,000 in non-protein blocking solution, 1 h, 20°C). RPLP0 was used as a loading control (1:2000 in TBST, 5% BSA at 4°C overnight; Lifespan Biosciences) conjugated with horse radish peroxidase anti rabbit secondary antibody (1:10000 in1x non-protein solution, 1 h, RT; Cell signalling). The chemiluminescence signal was detected by West Dura substrate (Pierce, Rockford, United States).

### Proliferation Assay

Proliferation of cells was performed by DAPI staining as described by (Ligasova and Koberna, 2019). Briefly, 800 C6 (w/o Δ5 or FL or Mut) and 800 C6 TAZ (w/o Δ5 or FL or Mut) cells were seeded per well of a 96 well plate. After incubation at 37°C and 5% CO_2_ for 96 h, the medium was aspirated, cells were fixed with 70% ethanol for 10 min at RT, and air-dried for 30 min. Cells were then incubated with 100 µl of 7.5 μM DAPI (Roth, Karlsruhe, Germany) in 150 mm NaCl and 20 mm Tris HCl pH 7.0 with constant shaking at 300 rpm for 30 min. Afterwards, cells were washed three times with 200 μl of washing buffer (2 mM CuSO_4_, 0.5 M NaCl, 20 mm citrate buffer pH 5.0, and 0.2% Tween 20) for 2 min each followed by rinsing with 100 μl of stabilization buffer (20 mm Tris HCl and 150 mm NaCl) to maintain the pH at 7.0, approximately. The stabilization buffer was then replaced by 120 μl of elution buffer (20 mm Tris HCl pH 7.0 and 2% SDS) and the plate was incubated at RT with constant shaking (300 rpm) for 15 min. Finally, 100 μl of the elution lysate were transferred to a clean black 96 well plate (Greiner Bio-one GmbH, Germany), and fluorescence intensity (RFI) was measured at 470/570 nm wavelength using a plate reader (Infinite F200 Microplate reader, Tecan).

### Senescence Associated β-Galactosidase Staining

Senescent cells were determined using Senescence β-galactosidase staining kit according to manufacturer protocol (Cell signalling Technology). Briefly, C6 and C6 TAZ cells were seeded in a 6 well plate at a density of 0.3 and 0.6 M cells. After incubation at 37°C and 5% CO_2_ the growth media was aspirated and rinsed one time with 1× PBS. Later, cells were fixed with 1 ml of 1× fixative solution (dilute 10× fixative solution in distilled water). After fixation rinse again 2 times with 1× PBS. Further, stain the cells with 1 ml of β galactosidase staining solution pH 6.0 (930 µl of 1× staining solution, 10 µl of 100 × solution A, 10 µl of 100 × solution B, 10 µl 20 mg/ml X-gal stock solution) and incubate overnight at 37°C. Finally, after 24 h β-gal positive cells were seen under the microscope.

### Cellular Cholesterol Content

The cholesterol content of the different C6 cell populations has been determined by means of the Cholesterol/Cholesteryl Ester Assay-Kit (ab102515, abcam) exactly following the recommended protocol.

### Statistical Analysis

Statistical analysis was performed using Graph Pad Prism 8 (GraphPad Inc., La Jolla United States). Data is shown as box plot with median, quartile and interquartile range. Comparison of two groups was performed using unpaired *t* test. *p* < 0.05 is considered to show significant changes.

## Results

### TAZ Deficiency Alters Oxylipin Profiles

**FIGURE 1:**
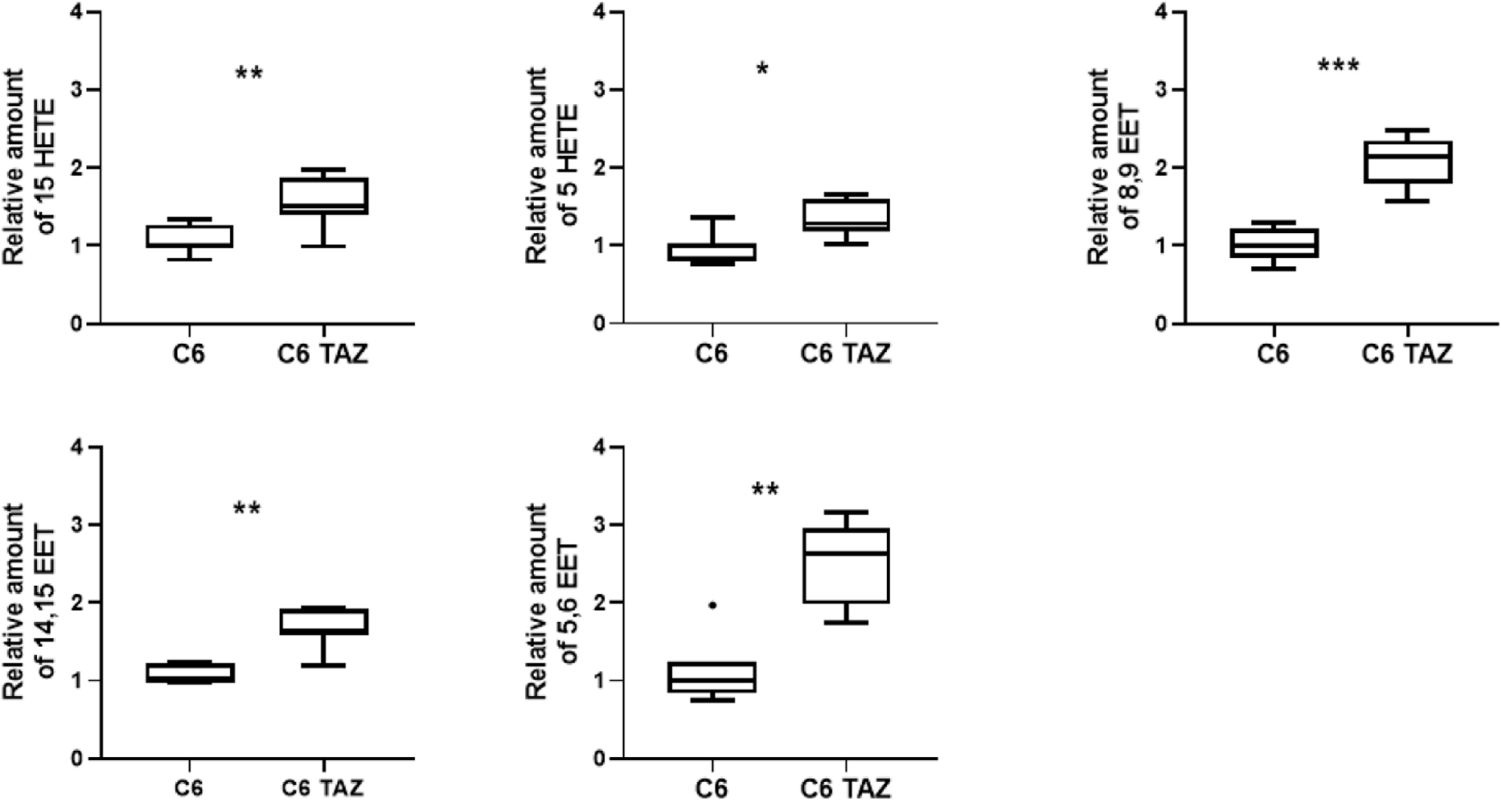
Tafazzin deficiency alters oxylipin levels in C6 cells: Effect of tafazzin knockout on cellular lipids, oxylipinswas determined by HPLC-MS/MS. The Data analysis was performed with Agilent Mass Hunter Qualitative and Quantitative Analysis software (version B.08.00 for both). For all detected oxylipins, a relative quantification was done by normalizing the area of the metabolite signal to the area of the signal of the internal standard compound (relative amount). Data is represented as median,quartile and interquartile range for *n* = 4.

It is well established that tafazzin-deficiency alters cardiolipin composition in patients and various experimental models (Joshi et al., 2009; Lu et al., 2016; Pu, 2022). This applies also to C6 rat glioma cells where the knock-out of tafazzin has been shown previously to alter CL composition and, consequently, to compromise mitochondrial respiration, and cellular proliferation (Murke et al., 2016; Ohlig et al., 2018; Gurtler et al., 2019; Schild et al., 2020). Alterations of the cellular lipid profile that arise from tafazzin-deficiency are not limited to the cardiolipin composition, but also relate to fatty acids, for example, be they free or bound, e.g., to other phospholipids. We show here, that the knock-out of tafazzin in C6 cells is associated with the dysregulation of another class of cellular lipids, namely the oxylipins. In C6-TAZ cells we measured higher amounts of 15-HETE [C6-TAZ: 1.52 (1.40; 1.88) vs. C6: 1.00 (0.96; 1.28); *p* = 0.0076], 5-HETE [C6-TAZ: 1.28 (1.18; 1.61) vs. C6: 1.00 (0.79; 1.04); *p* = 0.0175], 8,9 EET [C6-TAZ: 2,14 (1,80; 2.35) vs. C6: 1.00 (0.85; 1.22); *p* = 0.0006], 14,15 EET [C6-TAZ: 1.92 (1.60; 1.92) vs. C6: 1.00 (0.99; 1.23); *p* = 0.0023], and 5,6 EET [C6-TAZ: 2.63 (1.98; 2.96) vs. C6: 1.00 (0.84; 1.24); *p* = 0.0012] (Figure 1).

### TAZ Deficiency Alters Gene Expression

In this study, the unexpected finding is reported that TAZ deficiency also provokes substantial alterations in gene expression in rat glioma C6 cells. By applying RNA microarray analysis, gene expression of wild-type and tafazzin-deficient C6 cells was compared.

Microarray-based mRNA profiling revealed that in response to the knock-out of tafazzin, there are substantial (≥2fold) changes in transcript levels in rat C6 glioma cells (C6-TAZ vs. C6: 542 genes up-regulated, 484 down-regulated; a total of 1,026 genes differentially expressed). A ≥4fold change in expression levels in C6-TAZ cells could be observed for 73 genes compared to C6: 42 genes were up-regulated, 31 genes down-regulated. In Table 2 the top 15 up- and down-regulated genes are summarized. A complete list of the microarray results is given in Online Supplementary Table S1.

**TABLE 2:** Differentially expressed genes in taffazin-deficient C6 rat glioma cells (15 top up-regulated and 15 Top down-regulated genes)

Gene Symbol	Description	Fold Change
MPZ	myelin protein zero	53.36
FMOD	fibromodulin	38.87
OLFML2A	olfactomedin-like 2A	27.22
TGFBI	transforming growth factor. beta induced	18.42
ISLR	immunoglobulin superfamily containing leucine-rich repeat	16.7
**PLP1**	**proteolipid protein 1**	**15.95**
FOS	FBJ osteosarcoma oncogene	14.96
ENPP2	ectonucleotide pyrophosphatase/phosphodiesterase 2	10.92
ADH7	alcohol dehydrogenase 7 (class IV). mu or sigma polypeptide	9.23
RGR	retinal G protein coupled receptor	7.66
PMEL	premelanosome protein	7.62
FGFR3	fibroblast growth factor receptor 3	7.23
ART3	ADP-ribosyltransferase 3	6.94
ZFP36	zinc finger protein 36	6.84
**CXCL1**	**chemokine (C-X-C motif) ligand 1**	**6.84**
NEK2	NIMA-related kinase 2	−4.84
ACAT2	acetyl-CoA acetyltransferase 2	−4.89
FDPS	farnesyl diphosphate synthase	−5.00
STARD4	StAR-related lipid transfer (START) domain containing 4	−5.06
IDI1	isopentenyl-diphosphate delta isomerase 1	−5.20
MVD	mevalonate (diphospho) decarboxylase	−5.38
MURC	muscle-related coiled-coil protein	−5.43
LOC500300	similar to hypothetical protein mgc6835; found in mitochondrion (ortholog) and interacts with 1-naphthyl isothiocyanate (ortholog) and 17beta-estradiol (ortholog) and 2,3,7,8-tetrachlorodibenzodioxine (ortholog)	−5.56
TM7SF2	transmembrane 7 superfamily member 2	−5.77
**MVK**	**mevalonate kinase**	−**5.88**
**CPM**	**carboxypeptidase M**	−**6.13**
BRINP1	bone morphogenetic protein/retinoic acid inducible neural-specific 1	−6.26
HSD17B7	hydroxysteroid (17-beta) dehydrogenase 7	−6.79
OLAH	oleoyl-ACP hydrolase	−7.08
ACSS2	acyl-CoA synthetase short-chain family member 2	−7.35

Differential expression of genes indicated in bold has been confirmed by RT-qPCR (Figure)

### Validation of Transcriptome Data

**FIGURE 2:**
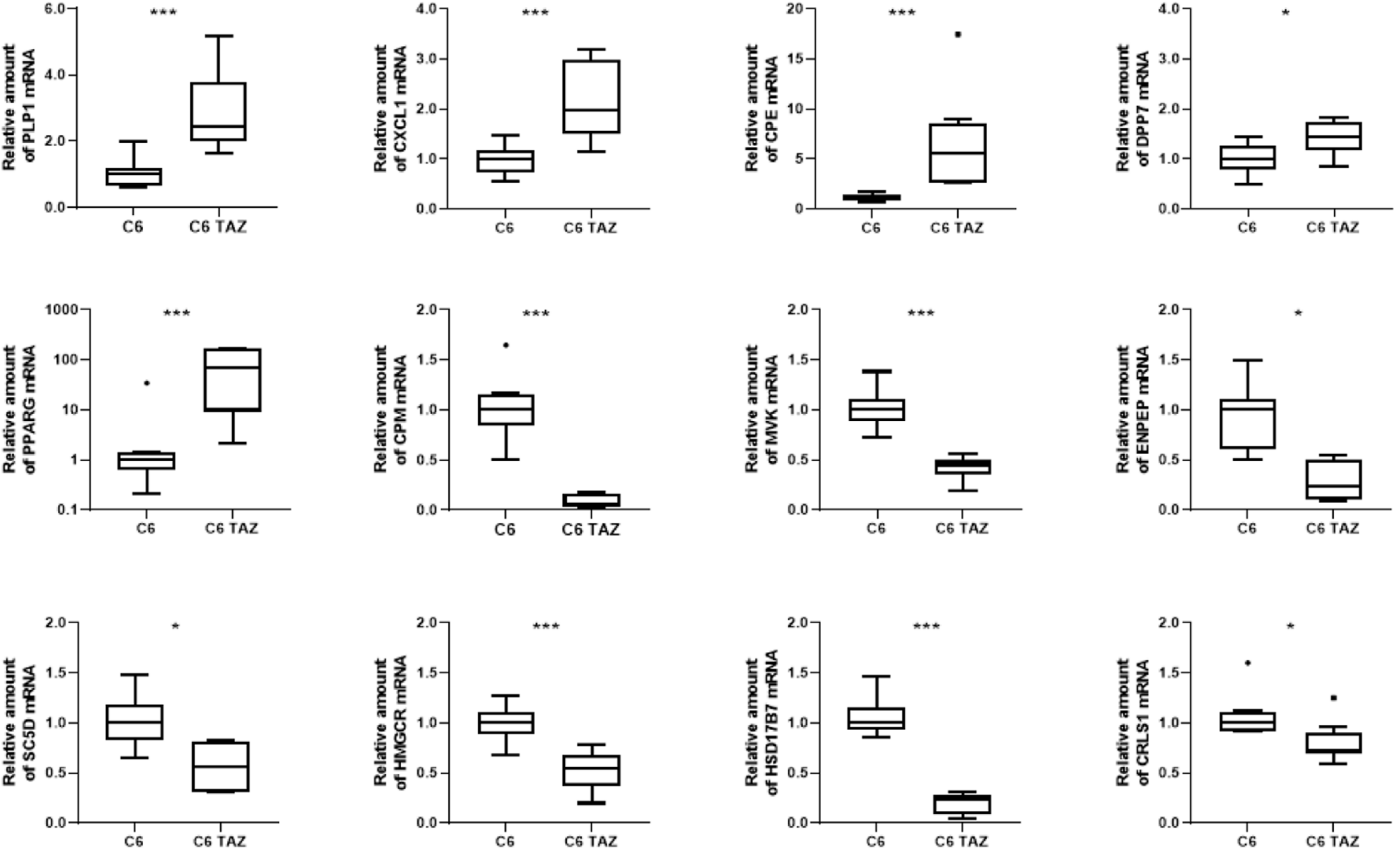
Tafazzin deficiency alters gene expression in C6 cells: Quantification levels of indicated genes in C6 and C6 TAZ cells was preformed and the amount of mRNA was determined by using RT-qPCR and normalized to RPL13a. Data is given as boxplot with median, quartile, and interquartile range (*p < 0.05; **p < 0.01; ***p < 0.001). Outliers are indicated using Tukey method. *n* = 8.

Microarray-based gene expression data were subsequently confirmed by RT-qPCR for selected targets. As shown in Figure 2, validation experiments fully confirmed the differential gene expression of TAZ-regulated genes in C6 glioma cells. In detail, tafazzin-deficient C6-TAZ cells contain higher amounts of the mRNAs for PLP1 [2.44 (2.00; 3.81)] vs. [1.00 (0.66; 1.20); *p* = 0.0006], CXCL1 [1.97 (1.50; 3.00)] vs. [1.00 (0.72; 1.18); *p* = 0.0012], which were among the 15 up-regulated genes in the microarray analysis (Figure 2). In addition, higher expression levels of further selected genes that showed ≥2-fold expression changes in the microarray analysis could be verified by RT-qPCR: mRNA amounts for CPE [5.55 (2.67; 8.54)] vs. [1.00 (0.87; 1.39); *p* = 0.0002)], DPP7 [1.44 (1.16; 1.73) vs. [1.00 (0.79; 1.26); *p* = 0.0281], and PPARG [69.74 (9.21; 162.5)] vs. [1.00 (0.61; 1.39); *p* = 0.0019] were increased in tafazzin-deficient C6-TAZ cells. Results of transcriptome analysis identified CPM and MVK among the top-regulated genes showing decreased expression in TAZ-deficient cells. This finding could be validated by RT-qPCR for the top-regulated genes, CPM [0.065 (0.026; 0.162)] vs. [1.00 (0.84; 1.15); *p* = 0.0002] and MVK [0.45 (0.36; 0.50)] vs. [1.00 (0.89; 1.12); *p* = 0.0002], as well as for selected examples of genes the expression of which showed a ≥2-fold decrease: ENPEP [0.24 (0.095; 0.50)] vs. [1.00 (0.61; 1.10); *p* = 0.0121], SC5D [0.57 (0.31; 0.81)] vs. [1.00 (0.83; 1.19); *p* = 0.0104], HMGCR [0.54 (0.37; 0.67)] vs. [1.00 (0.88; 1.11); *p* = 0.0006], HSD17B7 [0.24 (0.09; 0.28)] vs. [1.00 (0.93; 1.16); *p* = 0.0002], and CRLS1 [0.72 (0.69; 0.90)] vs. [1.00 (0.92; 1.11); *p* = 0.0281] (Figure 2).

### Oxylipins Contribute in Regulation of TAZ-Dependent Gene Expression

**FIGURE 3:**
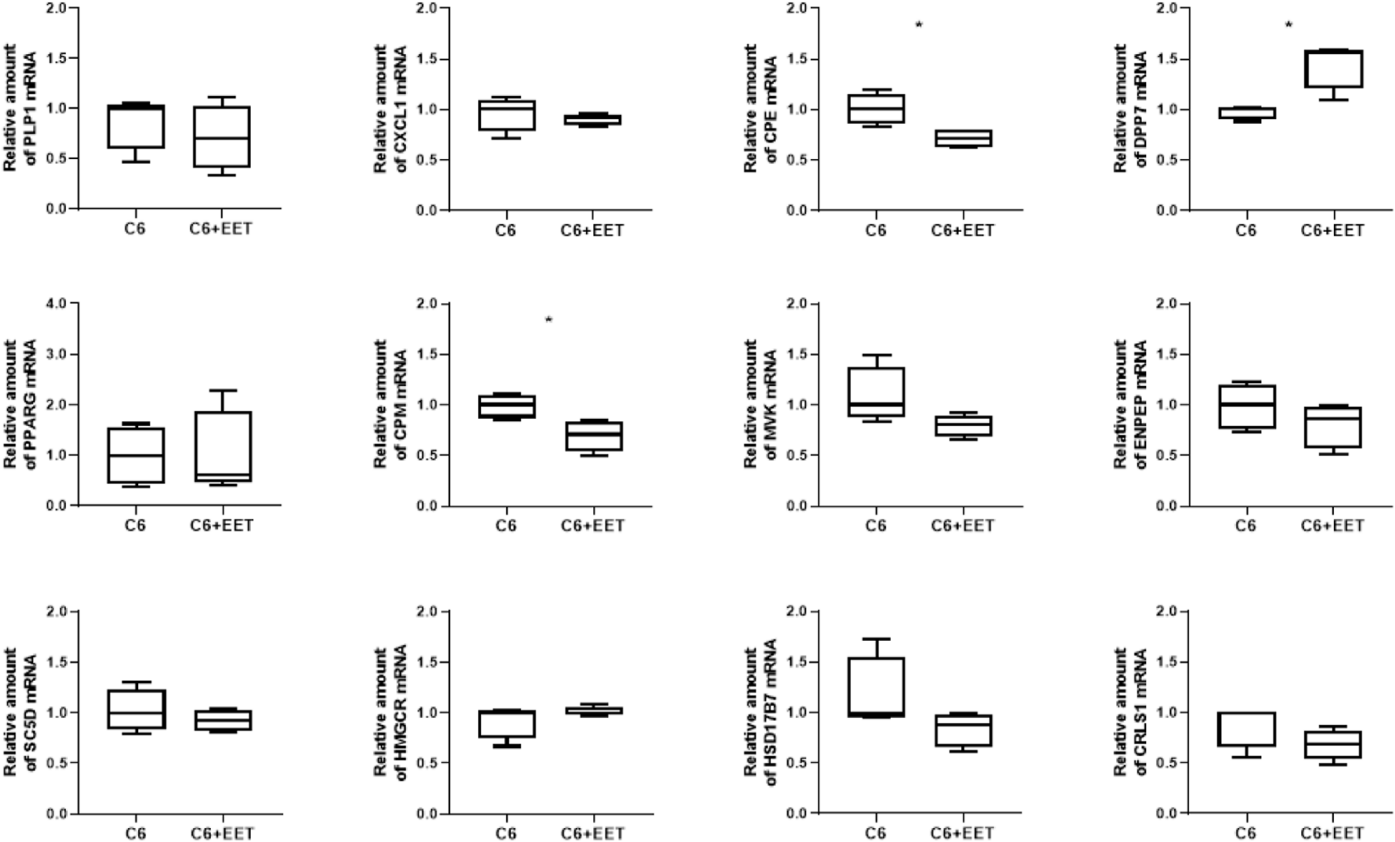
Oxylipins regulates TAZ dependent gene expression in C6 cells: Relative gene expression of C6 cells treated with ethanol (control) and C6 cells with 3 µM EET incubated at 37°C for 24 h. The amount of Mrna is determined by qpcr that is normalized to RPL13a. Data is represented as boxplot with median, quartile and interquartile range. Asterisks represent significant change.

Oxylipins are considered as ligands for PPARs (Cowart et al., 2002; Flores et al., 2005; Liu et al., 2005; Oh et al., 2009; Korbecki et al., 2019; Basilotta et al., 2022) and, thus, it was hypothesized that elevated concentrations of distinct lipoxins observed after knock-out of tafazzin contribute to TAZ-dependent changes in gene expression in C6 cells. Therefore, C6 cells were exposed to 8,9 EET, which showed the greatest increase in concentration under tafazzin knock-out in C6 TAZ cells, at 3 µM concentration for 24 h and then expression of selected genes was determined by RT-qPCR. Consistent with both the results of the transcriptome analysis and its verification, increased concentrations of 8,9 EET were associated with decreased expression of CPM [0.71 (0.53; 0.83) vs. 1.00 (0.87; 1.10); *p* = 0.0286] but elevated mRNA amounts of DPP7 [1.56 (1.20; 1.59) vs. 1.00 (0.91; 1.02); *p* = 0.0286] (Figure 3). However, 8,9 EET at this concentration also reduced the mRNA amounts of CPE [0.72 (0.63; 0.81) vs. 1.00 (0.86; 1.15); *p* = 0.0286], which was found induced by TAZ-knock-out and the concomitant increase in 8,9 EET levels (Figure 3). Expression levels of PLP1, CXCL1, PPARG, MVK, SC5D, HMGCR, ENPEP, CRLS1, and HSD17B7 were not affected under the conditions applied.

### TF Binding Site Identification

Oxylipins, including HETEs and EETs, have been described as endogenous ligands of PPARs (Cowart et al., 2002; Flores et al., 2005; Liu et al., 2005; Oh et al., 2009; Korbecki et al., 2019; Basilotta et al., 2022). Furthermore, 12 out of the 12 genes that were differentially expressed in C6 TAZ cells as confirmed by RT-qPCR (shown in Figure 2), according to the eukaryotic promoter database (EPD) (Dreos et al., 2013) contained at least one putative PPARG or PPARA: RXRA binding site in their promoters (Online Supplementary Table S2; *p* < 0.001). Therefore, we hypothesized, that the dysregulation of lipid metabolism in general and that of oxylipins in particular, could contribute to TAZ-dependent changes in changes in gene expression via PPARs. Nevertheless, many different potential TF binding sites have been predicted for the datasets suggesting that no specific TF binding profile based solely on PPARs is present. From the microarray dataset we included 995 genes between a foldchange of 1 and −1 as background and used the 2-fold and 4-fold up- (2fold: 544 genes; 4fold: 41 genes) and down-regulated (2fold: 496 genes; 4fold: 31 genes) datasets as input for a TF binding site enrichment analysis. After removing not identified genes from the database by Ciiider we used our approach to compare the qualitative TF binding site occurrence with the quantitative TF binding site enrichment. For the 2-fold down- and upregulated list of genes the enriched TF binding profiles after removing the enriched background TF binding profiles were from HOXA5 and Pax2, which were occurring in ∼100% of all genes. Interestingly, in the 4-fold down- and upregulated list of genes only Pax2 was still enriched. In contrast, the binding profiles for FOS:JUN(var.2), Lhx4, NHLH1, RHOXF1, Sox5, TBX20 and ZNF354C were exclusively enriched in the 4fold downregulated list of genes (Supplementary Figure S1). For the upregulated genes the exclusively enriched TF binding profiles were FOS::JUN(var.2) (∼31% of sequences), JUN::JUNB (∼86% of sequences), LEF1 (∼9% of sequences), NR1H4 (∼51% of sequences) and SOHLH2 (∼37% of sequences) (Supplementary Figure S2).

### Canonical Pathways Altered by Knock-Out of Tafazzin

Ingenuity pathway analysis (IPA) identified Diseases and Functions affected by tafazzin knock-out. As shown in Supplementary Table S3, the top-regulated Diseases and Functions include [Carbohydrate Metabolism, Lipid Metabolism, Molecular Transport] and [Lipid Metabolism, Small Molecule Biochemistry, Vitamin and Mineral Metabolism]. For all the identified pathways indicated above, selected members could be verified by RT-qPCR. This is shown e.g., for the cholesterol biosynthesis pathway (Supplementary Figure S3) where four out of the 20 differentially expressed genes (MVK, HSD17B7, SC5D, and HMGCR; Table 3) have been validated (Figure 2).

**TABLE 3:** Up- and down-regulated genes in cholesterol biosynthesis superpathway

No	Gene symbol	Gene	C6	C6 TAZ	Fold change
1	TRIT1	Dimethylallyltransferase	8.89	9.14	1.19
2	PGGT1B	Geranyltransferase	11.65	11.85	1.15
3	**HSD17B7**	**3-keto-steroid reductase/17-beta-hydroxysteroid dehydrogenase 7**	**12.94**	**10.17**	−**6.79**
4	**MVK**	**mevalonate kinase**	**12.38**	**9.82**	−**5.88**
5	TM7SF2	Delta(14)-sterol reductase	12.36	9.83	−5.77
6	MVD	Diphosphomevalonate decarboxylase	9.11	6.68	−5.38
7	IDI1	Isopentenyl-diphosphate Delta-isomerase 1	13.29	10.92	−5.2
8	CYP51	Lanosterol 14-alpha demethylase	15.97	13.72	−4.75
9	NSDHL	Sterol-4-alpha-carboxylate 3-dehydrogenase, decarboxylating	12.46	10.25	−4.61
10	HMGCS1	Hydroxymethylglutaryl-CoA synthase	14.62	12.69	−3.83
11	FDFT1	Squalene synthase	14.79	12.87	−3.79
12	**SC5D**	**Lathosterol oxidase**	**14.66**	**12.8**	−**3.64**
13	LSS	Lanosterol synthase	12.36	10.52	−3.59
14	DHCR7	7-dehydrocholesterol reductase	12.6	10.83	−3.43
15	EBP	3-beta-hydroxysteroid-Delta(8),Delta(7)-isomerase (Cholestenol Delta-isomerase)	12.72	11.11	−3.05
16	DHCR24	Delta(24)-sterol reductase	15.25	13.64	−3.04
17	PMVK	Phosphomevalonate kinase	10.09	8.57	−2.87
18	**HMGCR**	**3-hydroxy-3-methylglutaryl-coenzyme A reductase**	**12.45**	**11.19**	−**2.4**
19	MSMO1	Methylsterol monooxygenase 1	16.36	15.17	−2.29
20	ACAT1	Acetyl-CoA acetyltransferase	11.43	10.82	−1.52

In the end we performed a TF binding site profile detection analysis to find common regulatory elements for the top-regulated genes of the cholesterol pathway specifically. Based on the promoter panel (Supplementary Figure S4) of the 20 involved genes we found binding sites for FOS:JUN(var.2), LHX9, MLX, MNX1, NKX2-5, NR1H4, Pax2, SOX15, Stat2, and TBX19 (Supplementary Figure S2). Many of these TFs have more than one putative binding site per sequence. For this reason, we performed an enrichment analysis of the promoters against the background set of none-affected genes and came to the same involved TFs involved in the cholesterol pathway.

### Upstream Regulator Analysis

**FIGURE 4:**
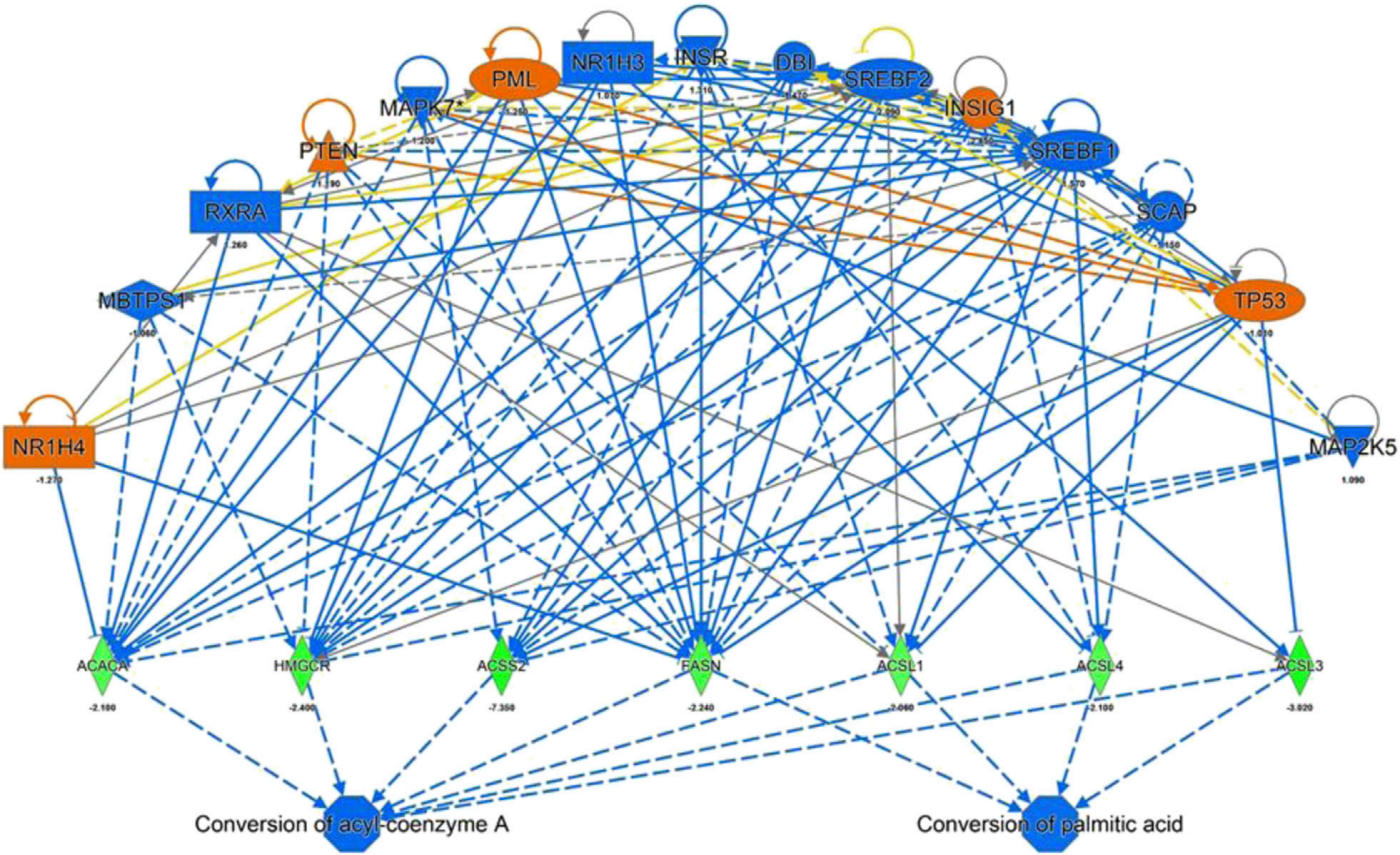
IPA Upstream Regulators Analysis for identification of upstream regulators that may be responsible for changes in gene expression observed for ACACA, HMGCR, ACSS2, FASN, ACSL 1,3 and 4 (predicted activation (orange) predicted inhibition (blue) of the regulators leads to a decreased expression (green)).

IPA Upstream Regulator Analysis was used to identify the upstream regulators that may be responsible for gene expression changes observed in the experimental dataset. Analysis was based on expected causal effects between upstream regulators and targets - the expected causal effects are derived from the literature compiled in the Ingenuity Knowledge Base (QIAGEN IPA; QIAGEN Inc.). As summarized in Figure 4 ACACA, HMGCR, ACSS2, FASN, ACSL1, 3 and 5 were identified as targets with a decreased gene expression compared to the control. These genes known to be inhibited by the following regulators: NR1H4, PTEN, PML, INSIG1, TP53 by a predicted activation and MBTPS1, RXRA, MAPK7, NR1H3, INSR, DBI, SREBF1/2, SCAP, MAP2K5 by a predicated inhibition of the regulators itself. Gene expression of HMGCR have been validated for a selected target gene (Figure 2) while binding sites for predicted regulators (e.g. NR1H4) have been confirmed using the TF binding site profile detection analysis (Supplementary Figure S2).

### Effect of TAZ Deficiency on Cellular Senescence

C6 TAZ cells showed significantly decreased levels of cell proliferation when compared to wild-type C6 cells (Gurtler et al., 2019). To evaluate if the development of cellular senescence contributes to this phenomenon β-galactosidase (SA-βGal) activity was determined in C6 and C6 TAZ cells. Indeed, C6 TAZ cells showed a more than 70fold increase in the fraction of senescent cells (C6 TAZ: 22.64 ŷ 6.90% β-Gal positive cells; versus C6: 0.32 ŷ 0.13% β-Gal positive cells; *p* = 0.029) (Figure 4A). Senescent cells exhibit a typical phenotype, senescence associated secretory phenotype (SASP). This is characterized by the expression/release of, among others, pro-inflammatory cytokines (IL6, IL1α), chemokines (IL8, CXCL1), proteases (MMPs), soluble factors like colony stimulating factors GM-CSF, G-CSF), and CDK inhibitors (p16, p21) (Ortiz-Montero et al., 2017; Cuollo et al., 2020). Here we demonstrate by RT-qPCR that mRNA amounts of CXCL1, IL6, and ILα were increased in C6 TAZ compared to C6 cells (CXCL1: 1.96[1.50; 3.00] vs. 1.00[0.72; 1.18]; *p* = 0.0012; IL6: 3.60[3.03;4.13] vs. 1.00[0.72;2.43]; *p* = 0.0286; and IL1α: 1.34[1.21;1.44] vs. 1.00[0.75;1.16]; *p* = 0.057) (Figure 4B).

### Re-Expression of Tafazzin in Tafazzin-Deficient C6 TAZ Cells

**FIGURE 5:**
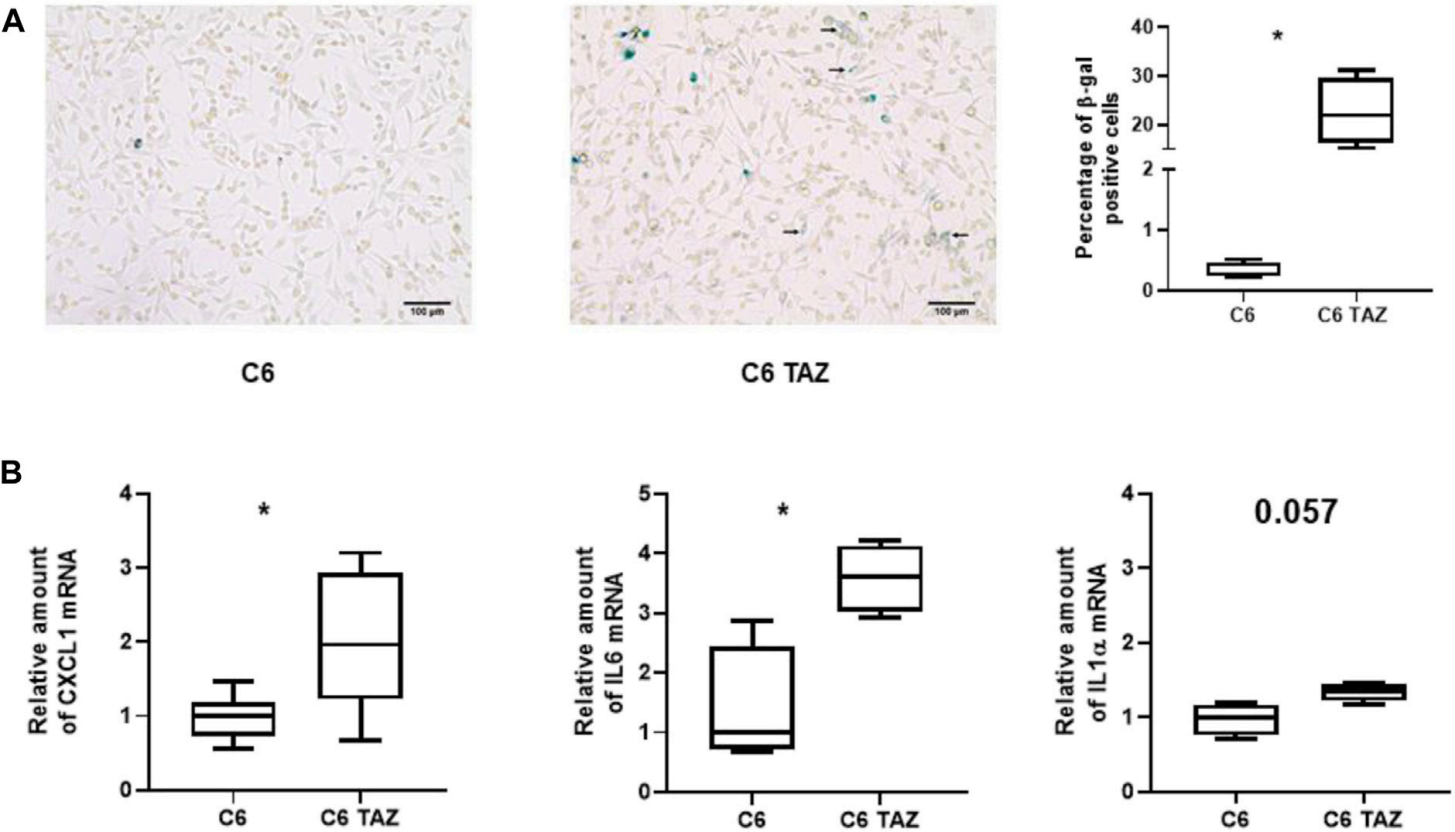
**(A)** Left: Microscopic images of β-gal positive cells (blue color) in C6 and C6 TAZ cells. Right: Percentage of β-gal positive cells is given as boxplot (* indicates *p* < 0.05). **(B)** Upregulation of SASP genes: Amounts of mrnas of CXCL1, IL6, and IL1α are elevated in C6 TAZ cells (mrna expression normalized to RPL13a; *n* = 4, *p* = 0.029).

Next, the tafazzin variants (wild-type full length, FL; H69L enzymatically dead mutant, Mut; and tafazzin lacking exon 5, Δ5) were re-expressed in C6 TAZ cells to elucidate, to what extent these variants are able to reverse tafazzin-knock-out-dependent changes in cellular functions. By transfection and subsequent selection, expression of the three tafazzin variants could be achieved in tafazzin-deficient C6-TAZ cells. As shown in Figure 5, all three tafazzin variants could be successfully expressed in C6 cells at a level by far exceeding that of wild-type C6 cells.

### Effects of TAZ Variants on CL-Composition

First of all, of course, it was analysed to what extent the re-expression of the different TAZ variants could reverse the massive changes in the cardiolipin composition caused by the knock-out of tafazzin in rat C6 cells. As summarized in Table 4, the tafazzin variants full length TAZ and Δ5 TAZ were able to reverse to a large extent the changes in the CL composition observed after TAZ KO. In contrast, and as expected, the mutant lacking transacylase activity (H69L; Mut), did not restore the CL-composition associated with the C6 wildtype phenotype. Of note, for 11 out of 17 individual CL-species (abundance < 0.1; Table 1) re-expression of FL tafazzin resulted in amounts which are closer to the C6 wild-type compared to the ones obtained by re-expressing the Δ5 variant. This also expressly applies to the content of oxidized cardiolipin [C6: 11 (9.46; 11.75); C6 TAZ Δ5: 13.60 (12.65; 14.81); C6 TAZ FL: 10.01 (8.62; 11.17); Table 4].

**TABLE 4:** Cardiolipin composition of C6 (w/o Δ5, FL and Mut) and C6 TAZ (w/o Δ5, FL and Mut) cells

Cardiolipin Composition	C6 [Q1:Q3]	C6 TAZ [Q1:Q3]	C6 TAZ+Δ5 [Q1:Q3]	C6 TAZ+FL [Q1:Q3]	C6 TAZ+Mut3 [Q1:Q3]	C6+Δ5 [Q1:Q3]	C6+FL [Q1:Q3]	C6+Mut3 [Q1:Q3]	C6 vs.C6 TAZ	C6 vs. C6+Δ5	C6 vs. C6+FL	C6 vs. C6+Mut3	C6 TAZ vs. C6 TAZ +Δ5	C6 TAZ vs. C6 TAZ +FL	C6 TAZ vs. C6 TAZ +Mut3	C6 vs.C6 TAZ+Δ5	C6 vs. C6 TAZ+FL	C6 vs. C6 TAZ+Mut3	C6+Δ5 vs. C6 TAZ+Δ5	C6+FLvs. C6 TAZ+FL	C6+Mut3 vs. C6 TAZ+ Mut3
(C18:2)4	0.95 [0.87 ; 1.19]	0.09 [0.08;0.18]	0.80 [0.70;1]	1.15 [1.04;1.26]	0.06 [0.05;0.07]	0.84 [0.73;1.0]	1.49 [1.21;1.68]	1.02 [0.91;1.17]	**	n.s	n.s	n.s	n.s	n.s	n.s	n.s	n.s	*	n.s	n.s	n.s
(C18:2)3/C18:1	2.68 [2.28;3.12]	0.13 [0.13;0.19]	2.03 [1.79;2.37]	2.27 [1.99;2.66]	0.07 [0.07;0.08]	1.82 [1.74;1.97]	3.85 [3.13;4.08]	3.46 [3.20;3.53]	****	n.s	n.s	n.s	**	**	n.s	n.s	n.s	****	n.s	n.s	***
(C18:2)2/(C18:1)2	8.40 [7.40;8.90]	0.52 [0.51;0.69]	6.05 [5.80;6.86]	5.83 [4.82;6.81]	0.84 [0.45;1.21]	6.42 [6.09;6.5]	8.90 [7.63;9.42]	8.09 [7.61;8.71]	****	n.s	n.s	n.s	****	****	n.s	*	*	****	n.s	n.s	****
C18:2/C18:1/(C16:0)2	0.53 [0.44;0.82]	0.84 [0.73;0.91]	0.51 [0.47;0.55]	0.86 [0.75;0.99]	1.00 [0.86;1.08]	0.47 [0.42;0.5]	0.64 [0.56;0.86]	0.97 [0.73;1.20]	n.s	n.s	n.s	n.s	n.s	n.s	n.s	n.s	n.s	n.s	n.s	n.s	n.s
(C18:1)2/(C16:0)2	10.18 [9.83 ;14.05]	42.73 [39.34;45.09]	14.11 [12.63;15.66]	13.11 [10.15;17.00]	47.26 [42.12;52.78]	9.42 [7.91;11.3]	7.59 [7.00;11.60]	17.81 [15.68;18.75]	****	n.s	n.s	n.s	****	****	n.s	n.s	n.s	****	n.s	n.s	****
(C18:2)3/C16:1	0.84 [0.79;1.00]	0.06 [0.04;0.09]	0.63 [0.55;0.70]	0.93 [0.75;1.10]	0.02 [0.02;0.02]	0.71 [0.60;0.87]	1.45 [1.10;1.77]	1.04 [0.88;1.30]	****	n.s	n.s	n.s	*	***	n.s	n.s	n.s	****	n.s	n.s	****
(C18:2)2/C18:1/C16:1 or (C18:2)3/C16:0	5.60 [4.87;6.10]	0.37 [0.30;0.46]	3.96 [3.89;4.32]	4.88 [3.71;5.86]	0.14 [0.11;0.16]	4.39 [3.96;6.45]	7.90 [6.80;8.54]	5.46 [4.87;6.83]	****	n.s	n.s	n.s	***	***	n.s	n.s	n.s	****	n.s	n.s	****
(C18:1)2/C18:2/C16:1 or (C18:2)2/C18:1/C16:0	17.76 [13.66;18.24]	1.17 [0.99;1.50]	13.47 [11.25;14.27]	15.12 [12.26;17.10]	0.77 [0.71;0.89]	16.37 [15.80;17.6]	19.10 [16.79;19.55]	13.77 [11.15;16.79]	****	n.s	n.s	n.s	****	****	n.s	n.s	n.s	****	n.s	n.s	****
(C18:1)2/C18:2/C16:0	4.83 [4.54;5.43]	1.78 [1.73;1.97]	4.44 [4.35;4.63]	4.85 [4.82;5.02]	1.16 [1.46;1.76]	4.43 [4.27;4.87]	4.84 [4.67;4.99]	5.72 [4.97;6.26]	****	n.s	n.s	n.s	****	****	n.s	n.s	n.s	****	n.s	n.s	****
(C18:1)3/C16:0	30.77 [27.44;31.64]	47.79 [44.05;51.52]	38.04 [36.97;38.98]	35.70 [31.94;39.51]	46.69 [41.83;51.19]	37.33 [35.23;37.99]	24.26 [22.42;28.97]	30.17 [26.37;32.04]	****	n.s	n.s	n.s	*	**	n.s	*	n.s	***	n.s	n.s	**
(C18:1)2/(C18:3)2	1.33 [1.12;1.66]	0.68 [0.51;0.83]	1.44 [1.19;1.56]	1.76 [1.52;2.00]	0.62 [0.50;0.74]	1.56 [1.23;1.83]	2.15 [1.81;2.30]	1.38 [1.14;1.62]	***	n.s	n.s	n.s	**	****	n.s	n.s	n.s	**	n.s	n.s	*
(C18:1)3/C18:2	10.87 [9.17;11.65]	1.18 [1.09;1.63]	9.41 [7.46;11.19]	9.29 [7.68;10.94]	0.80 [0.74;0.84]	11.60 [11.07;12.12]	11.27 [10.17;11.43]	8.36 [8.04;9.15]	****	n.s	n.s	n.s	****	****	n.s	n.s	n.s	****	n.s	n.s	****
(C18:2)3/C20:4	0.18 [0.14;0.24]	0.04 [0.03;0.09]	0.16 [0.12;0.23]	0.25 [0.22;0.28]	0.01 [0.01;0.02]	0.14 [0.12;0.19]	0.35 [0.25;0.43]	0.16 [0.14;0.20]	n.s	n.s	n.s	n.s	n.s	n.s	n.s	n.s	n.s	n.s	n.s	n.s	n.s
(C18:2)3/C20:3 o. (C18:2)2/C18:1/C20:4	0.46 [0.39;0.53]	0.03 [0.03;0.06]	0.3 [0.25;0.32]	0.55 [0.50;0.58]	0.02 [0.01;0.02]	0.29 [0.25;0.31]	0.70 [0.53;0.84]	0.55 [0.53;0.58]	***	n.s	n.s	n.s	n.s	**	n.s	n.s	n.s	***	n.s	n.s	**
(C18:2)3/C20:2 o. (C18:2)2/C18:1/C20:3	0.94 [0.81;1.02]	0.07 [0.05;0.13]	0.88 [0.86;0.92]	1.13 [1.07;1.19]	0.02 [0.02;0.03]	0.78 [0.69;1.03]	1.40 [1.16;1.56]	1.14 [1.05;1.24]	**	n.s	n.s	n.s	n.s	n.s	n.s	n.s	n.s	*	n.s	n.s	n.s
(C18:2)2/C18:1/C20:2	1.36 [1.28;1.61]	0.09 [0.07;0.12]	1.16 [0.99;1.45]	1.32 [1.15;1.49]	0.04 [0.03;0.05]	1.24 [1.11;1.40]	1.76 [1.40;1.97]	1.49 [1.29;1.71]	****	n.s	n.s	n.s	****	****	n.s	n.s	n.s	****	n.s	n.s	****
(C18:2)3/C20:0 or (C18:1)2/C18:2/C20:2	0.93 [0.79;1.02]	0.09 [0.08;0.37]	1.20 [0.98;1.39]	0.85 [0.71;0.97]	0.05 [0.04;0.07]	1.10 [0.88;1.32]	1.06 [0.82;1.25]	0.94 [0.70;1.17]	n.s	n.s	n.s	n.s	n.s	n.s	n.s	n.s	n.s	n.s	n.s	n.s	n.s
C22:6/(C18:2)3	0.01 [0.01;0.02]	0.01 [0.01;0.03]	0.04 [0.02;0.08]	0.01 [0.01;0.01]	0.01 [0.01;0.01]	0.01 [0.01;0.08]	0.01 [0.00;0.02]	0.01 [0.01;0.02]	n.s	n.s	n.s	n.s	n.s	n.s	n.s	n.s	n.s	n.s	n.s	n.s	n.s
C22:6/(C18:2)2/C18:1	0.01 [0.00;0.01]	0.00 [0.00;0.01]	0.01 [0.01;0.02]	0.01 [0.01;0.01]	0.00 [0.00;0.00]	0.01 [0.01;0.01]	0.02 [0.01;0.02]	0.00 [0.00;0.00]	n.s	n.s	n.s	n.s	n.s	n.s	n.s	n.s	n.s	n.s	n.s	n.s	n.s
CL total (ug/mg protein)	10.53 [3.08;14.09]	16.07 [6.33;20.32]	11.69 [9.94;19.64]	2.43 [2.33;2.59]	2.45 [2.27;2.72]	9.78 [8.12;16.51]	2.82 [2.50;3.09]	1.18 [1.09;1.24]	n.s	n.s	n.s	n.s	n.s	*	*	n.s	n.s	n.s	n.s	n.s	n.s
Oxidized CL	11 [9.46;11.75]	20.42 [15.54;24.69]	13.60 [12.65;14.81]	10.01 [8.62;11.17]	42.41 [33.11;47.77]	10.68 [10.22;12.19]	8.99 [8.47;9.75]	11.35 [11.29;11.44]	****	n.s	n.s	n.s	*	***	n.s	n.s	n.s	n.s	n.s	n.s	n.s
MLCL1 (C18:2)3 (µg/mg protein)	0.01 [0.00;0.03]	0.01 [0.0;0.04]	0.01 [0.01;0.03]	0.00 [0.00;0.00]	0.00 [0.00;0.00]	0.02 [0.01;0.04]	0.00 [0.0;0.0]	0.00 [0.00;0.00]	n.s	n.s	n.s	n.s	n.s	n.s	n.s	n.s	n.s	n.s	n.s	n.s	n.s
MLCL2 (% of MLCL1)	427.26 [352.78;450.57]	336.81 [254;382.70]	310.94 [172.95;453.97]	513.7 [408.46;583.18]	475 [416.2;572.2]	241.58 [163.09;278.63]	422.27 [371.44;445.76]	606.31 [580;620]	n.s	n.s	n.s	n.s	n.s	n.s	n.s	n.s	n.s	n.s	n.s	n.s	n.s

### Effects of TAZ Variants on Cellular Proliferation

**FIGURE 6:**
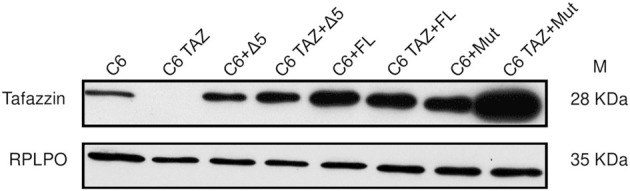
Re-expression of human Δ5, rat full length tafazzin (FL) and enzymatically dead mutant (Mut) in C6 and C6 TAZ knockout cells is confirmed by immunoblot using mouse anti tafazzin antibody. RPLPO is used as a loading control.

Next, it was analysed whether the re-expressed TAZ variants can reverse the marked reduction in cellular proliferation that has been observed after TAZ knock-down or knock-out, respectively. As summarized in Figure 6, C6 TAZ cells exhibit a substantial decrease in proliferation when compared to C6 wild-type cells [C6 TAZ: 0.28 (0.27; 0.36) vs. C6: 1.00 (0.79; 1.16), *p* < 0.001]. For control purposes, wild-type C6 cells were also transfected with the three TAZ variants. The stable expression of none of the three variants caused any significant change in cell proliferation compared to C6 wild-type, although there was a marginal increase upon transfection of the Δ5 variant (*p* = n.s.). Expression of the full-length rat tafazzin in C6 TAZ cells increased proliferation of C6 TAZ cells to the level of wild-type C6 cells [C6 FL: 1.10 (0.88; 1.17); C6 TAZ FL: 0.94 (0.76; 0.93); *p* = n.s.]. In contrast, and as expected, the expression of an enzymatically inactive mutant (H69L, Mut) failed to restore C6 proliferation [C6 Mut: 0.87 (0.79; 0.93) vs. C6 TAZ Mut: 0.31 (0.27; 0.38); *p* = 0.002]. Surprisingly, also the re-expression of the Δ5 variant did not lead to the restoration of the “wild-type” proliferation of C6 cells [C6 Δ5: 1.148 (0.93; 1.56) vs. C6 TAZ Δ5: 0.49 (0.40; 0.59); *p* < 0.0001] (Figure 6).

### Effects of TAZ Variants on Gene Expression

Finally, it was analysed to what extent the changes in gene expression resulting from down-regulation or lack of tafazzin in C6 cells could be normalized by re-expressing different tafazzin variants in C6 TAZ cells.

**FIGURE 7:**
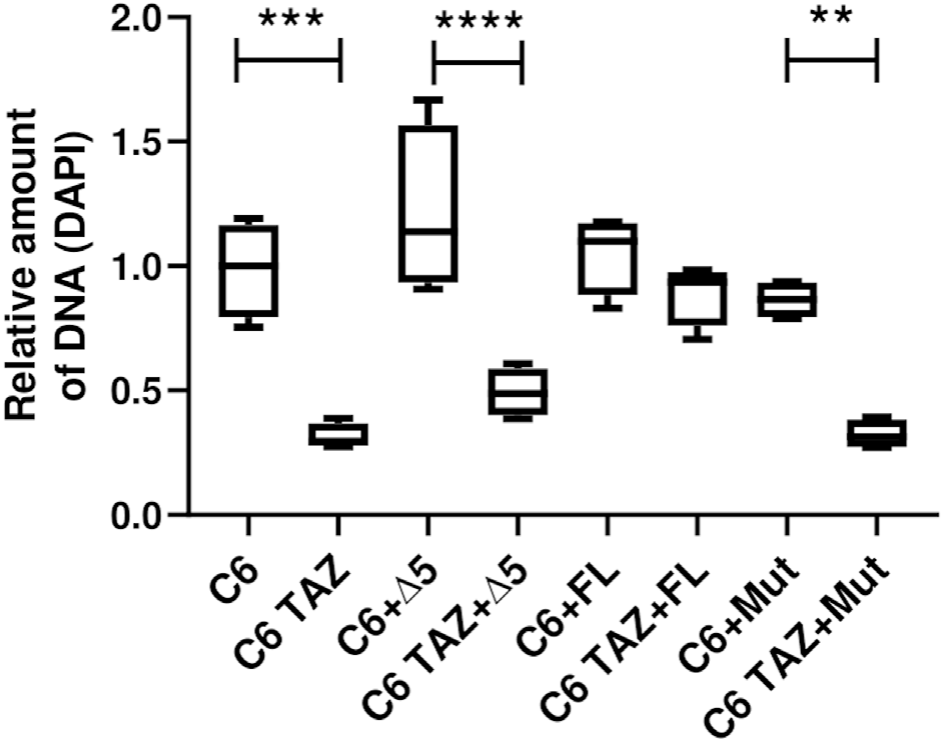
Proliferation was confirmed using DAPI stain. Density of 800 C6 and C6 TAZ cells (w/o Δ5, FL, Mut) were seeded in a 96 well plate. Cells were incubated for 96 h then fixed with ethanol and later stained with DAPI (7.5 µM). After subsequent washing steps the fluorescence intensity was measured at 450/570 nm wavelength. *n* = 4 biological replicates (C6 w/o Δ5, FL and Mut) and *n* = 4 biological replicates (C6 TAZ w/o Δ5, FL and Mut) is given as a boxplot with median, quartile and interquartile range.

**FIGURE 8:**
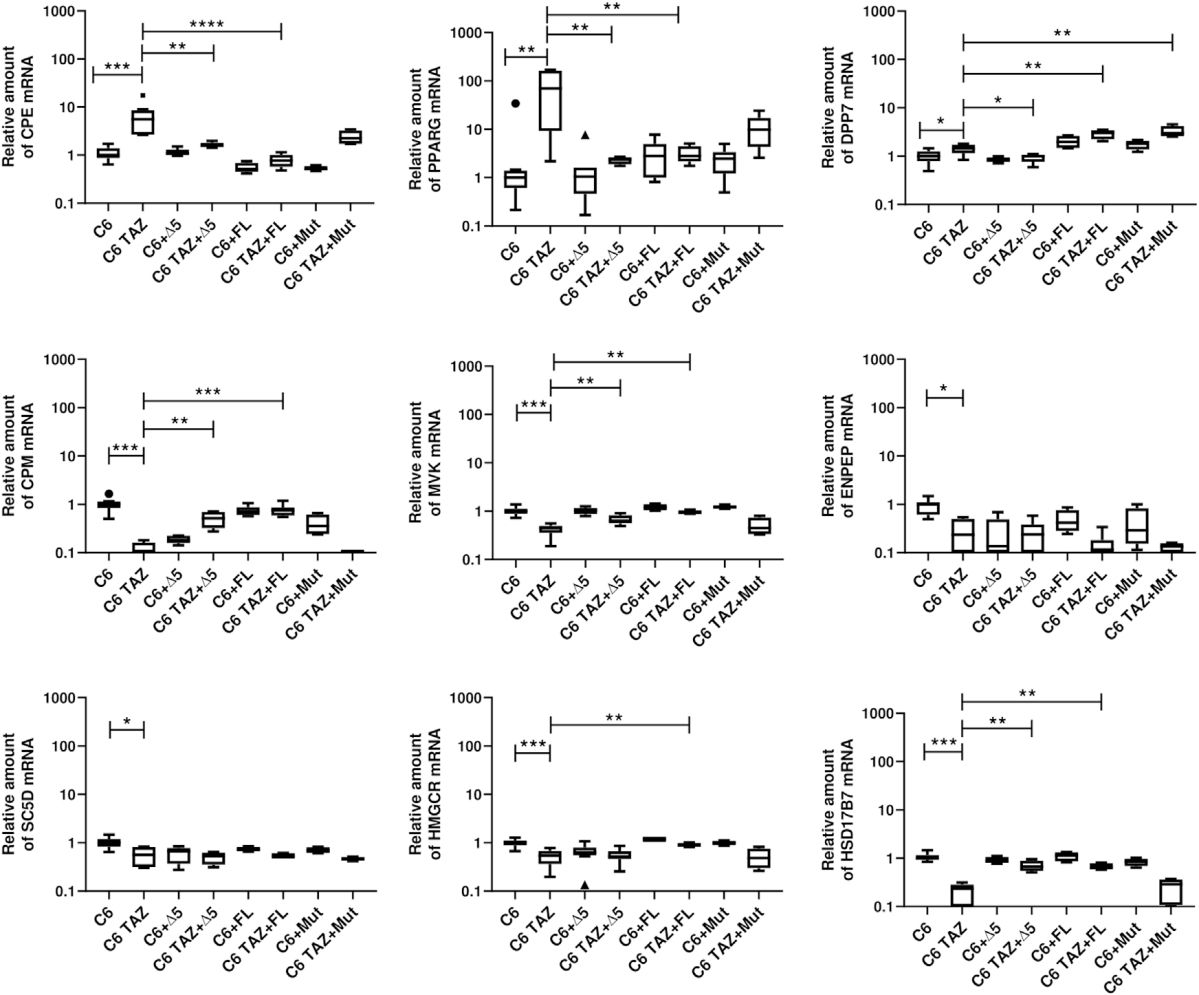
Relative mRNA amount of indicated genes in C6 and C6 TAZ (w/o Δ5, FL, Mut) is determined by RT-qPCR and normalized to RPLP13a. Data is represented as boxplot with median, quartile and interquartile range.

As shown in Figure 7, re-expression of FL-TAZ and Δ5-TAZ variants in C6-TAZ cells reversed the tafazzin-knock-out-dependent changes in gene expression to a large extent. In detail, FL-TAZ was able to reverse the tafazzin knock-out-dependent increase in mRNA expression of CPE [C6: 1.00 (0.87; 1.20); C6-TAZ: 5.55 (2.67; 8.53); TAZ-FL: 0.77 (0.57; 0.76); *p* < 0.0001 C6-TAZ vs. FL-TAZ] and of PPARG [C6: 1.00 (0.61; 1.39); C6-TAZ: 69.74 (9.21; 162.50); TAZ-FL: 2.80 (2.16; 4.48); *p* < 0.01 C6-TAZ vs. FL-TAZ]. Accordingly, CPM, MVK, HMGCR, HSD17B7, genes the mRNA amounts of which were found to be significantly down-regulated in response to tafazzin knock-out, were significantly increased by re-expression of FL-TAZ [CPM: C6: 1.00 (0.84; 1.15); C6-TAZ: 0.06 (0.03; 0.16); TAZ-FL: 0.76 (0.59; 0.87); *p* < 0.001 C6-TAZ vs. FL-TAZ; MVK [C6: 1.00 (0.89; 1.12); C6-TAZ: 0.45 (0.36; 0.50); TAZ-FL: 1.20 (1.04; 1.40); *p* < 0.01 C6-TAZ vs. FL-TAZ; HMGCR: C6: 1.00 (0.88; 1.11); C6-TAZ: 0.54 (0.37; 0.67); TAZ-FL: 0.89 (0.83; 0.99); *p* < 0.01 C6-TAZ vs. FL-TAZ; HSD17B2: C6: 1.00 (0.92; 1.16); C6-TAZ: 0.24 (0.09; 0.280); TAZ-FL: 0.66 (0.59; 0.79); *p* < 0.01 C6-TAZ vs. FL-TAZ] (Figure 8). However, FL-TAZ re-expression in C6-TAZ cells was not able to normalize decreased amounts of SC5D and ENPEP mRNA and even further elevated levels of DPP7 mRNA (Figure 8) [SC5D: C6: 1.00 (0.83; 1.19); C6-TAZ: 0.57 (0.31; 0.81); TAZ-FL: 0.53 (0.47; 0.61); *p* = n.s. C6-TAZ vs. FL-TAZ; ENPEP [C6: 1.00 (0.61; 1.10); C6-TAZ: 0.24 (0.09; 0.50); TAZ-FL: 0.12 (0.07; 0.18); *p* = n.s. C6-TAZ vs. FL-TAZ; DPP7: C6: 1.00 (0.79; 1.26); C6-TAZ: 1.44 (1.16; 1.73); TAZ-FL: 3.04 (2.24; 3.46); *p* < 0.01 C6-TAZ vs. FL-TAZ].

Δ5-TAZ, when re-expressed in C6-TAZ cells, was a little less effective than FL-TAZ in restoring C6 wild-type gene expression. Although Δ5-TAZ did alter gene expression towards wild-type, these changes were substantially less pronounced for CPE, CPM, and MVK compared to FL-TAZ. Furthermore, Δ5-TAZ did not restore wild-type levels of HMGCR mRNA atall, but, in contrast to FL-TAZ, did normalize DPP7 mRNA levels (Figure 7) [HMGCR: C6: 1.00 (0.88; 1.11); C6-TAZ: 0.54 (0.37; 0.67); Δ5-TAZ: 0.52 (0.46; 0.67); *p* = n.s. C6-TAZ vs. Δ5-TAZ; DPP7: C6: 1.00 (0.79; 1.26); C6-TAZ: 1.44 (1.16; 1.73); Δ5-TAZ: 0.99 (0.76; 1.08); *p* < 0.05 C6-TAZ vs. Δ5-TAZ; CPE: C6: 1.00 (0.87; 1.40); C6-TAZ: 5.55 (2.67; 8.53); Δ5-TAZ: 1.59 (1.46; 1.81); *p* < 0.01 C6-TAZ vs. Δ5-TAZ; CPM: C6: 1.00 (0.84; 1.15); C6-TAZ: 0.06 (0.03; 0.16); Δ5-TAZ: 0.52 (0.32; 0.70); *p* < 0.01 C6-TAZ vs. Δ5-TAZ; MVK [C6: 1.00 (0.89; 1.12); C6-TAZ: 0.45 (0.36; 0.50); Δ5-TAZ: 0.64 (0.56; 0.82); *p* < 0.01 C6-TAZ vs. Δ5-TAZ. The Δ5 variant also restored mRNA expression of PPARG and HSD17B7 (PPARG: C6: 1.00 (0.61; 1.39); C6-TAZ: 69.74 (9.21; 162.50); Δ5-TAZ: 2.38 (1.89; 2.63); *p* < 0.01 C6-TAZ vs. Δ5-TAZ; HSD17B7C6: 1.00 (0.61; 1.39); C6-TAZ: 69.74 (9.21; 162.50); Δ5-TAZ: 2.38 (1.89; 2.63); *p* < 0.01 C6-TAZ vs. Δ5-TAZ]. As observed for FL-TAZ, Δ5-TAZ was not able to return mRNA amounts of SC5D and ENPEP to wild-type levels [SC5D: C6: 1.00 (0.83; 1.19); C6-TAZ: 0.57 (0.31; 0.81); Δ5-TAZ: 0.53 (0.35; 0.63); *p* = n.s. C6-TAZ vs. Δ5-TAZ; ENPEP: C6: 1.00 (0.61; 1.10); C6-TAZ: 0.24 (0.09; 0.50); Δ5-TAZ: 0.24 (0.05; 0.39); *p* = n.s. C6-TAZ vs. Δ5-TAZ].

As expected, re-expressing the enzymatically in active variant, Mut, did not lead to any restoration of gene expression associated with the C6 wild-type.

### Effects of TAZ Variants on Cellular Cholesterol Contents

**FIGURE 9:**
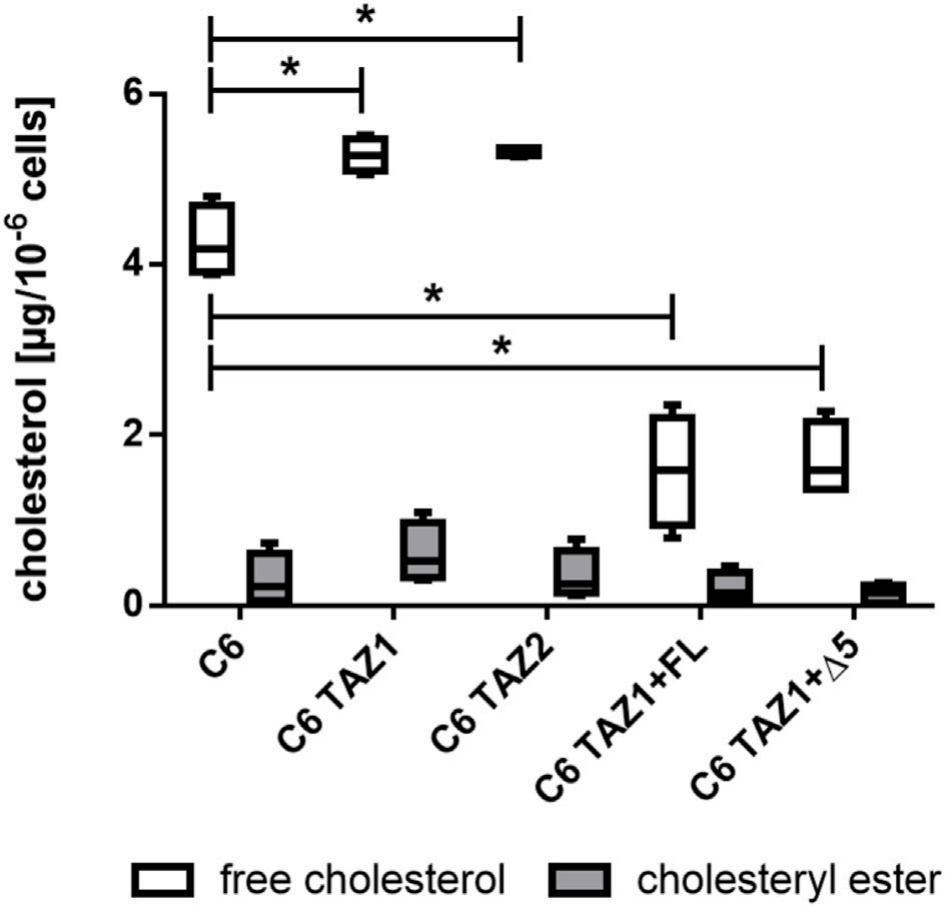
Cellular content of cholesterol (free and esterified) in C6, C6 TAZ cells, and in response to re-expression of full length tafazzin (C6 TAZ + FL) or tafazzin lacking exon 5 (C6 TAZ + Δ5). *n* = 4 biological replicates (data given as a boxplot with median, quartile and interquartile range).

As shown in Figure 9, the knock-out of tafazzin led to an increase in the amounts of cellular cholesterol [C6: 4.188 (4.80; 3.881) vs. C6 TAZ1: 5.285 (5.522; 5.057), *p* < 0.05; C6 vs. C6 TAZ2: 5.367 (5.367; 5.262), *p* < 0.05]. In C6 cells cholesterol is present mainly in its free form, only low levels of esterified cholesterol could be detected. Re-expression of either full length tafazzin (C6 TAZ + FL) or tafazzin lacking exon 5 (C6 TAZ + Δ5) substantially down-regulated the cells’ cholesterol content [C6 TAZ1: 5.285 (5.522; 5.057) vs. C6 TAZ1 + FL: 1.538 (2.347; 0.78), *p* < 0.05; C6TAZ1 vs. C6TAZ1 + Δ5: 1.591 (2.283; 1.365), *p* < 0.05].

## Discussion

It has been shown previously, that the knock-down or knock-out, respectively, of tafazzin in rat glioma C6 cells causes substantial changes in cellular and mitochondrial properties and functions. The changes affect CL composition and cellular fatty acid availability, mitochondrial respiration, mitochondrial ROS production, mitochondrial membrane fluidity, cellular elasticity and proliferation (Schild et al., 2012; Ohlig et al., 2018; Gurtler et al., 2019; Schild et al., 2020). It is now accepted that the cellular availability of fatty acids has a crucial impact on CL composition (Oemer et al., 2020; Oemer et al., 2021; Oemer et al., 2022). As shown in the present work, the knock-out of tafazzin in C6 cells also results in substantial changes in the concentration of oxylipins. Oxylipins can be formed by oxidation of cardiolipin acyl chains by ROS and subsequent release by e.g. phospholipase A2 (Jezek et al., 2010). In our recent publication we were able to demonstrate increased ROS formation in mitochondria of TAZ knockout cells (Gurtler et al., 2019). We assume that the altered cardiolipin composition of the inner mitochondria membrane in these cells impairs the function of the respiratory chain (mainly complex I) that causes increased ROS formation (Adam-Vizi, 2005; Wirth et al., 2016).

In their function as PPAR ligands, oxylipins such as 8,9-EET could potentially contribute to metabolic and gene expression changes observed in C6 TAZ cells. Accordingly, the administration of 3 µM 8,9-EET to C6 cells led to alterations in gene expression. Although this 3 µM 8,9-EET concentration exceeds plasma levels detected in humans and mice (Pozzi et al., 2005; Gao et al., 2012; Lee et al., 2013), the occurrence of higher tissue concentrations under certain pathophysiological conditions cannot be ruled out (Brenneis et al., 2011). However, the contribution of PPAR to tafazzin-dependent expression changes is supposed to be of marginal relevance only, since not PPAR binding sites but those for HOXA5 and Pax2 (which were occurring in ∼100% of all up-regulated genes) were found to be enriched in TAZ-regulated genes when applying more recent databases in transcriptome analysis. In support of a minor role of PPARs only, the addition of the PPARγ agonist, pioglitazone, at 10, 3 or 1 µM concentration failed to induce any changes in the mRNA levels of the TAZ-regulated genes shown in Figure 2.

It is further demonstrated that the knock-down of tafazzin in C6 cells provokes massive changes in gene expression. Defective CL remodelling has been associated with altered gene expression of HIF-1α previously (Chowdhury et al., 2018). Mechanistically, differential expression of HIF-1α could be explained by reduced ROS production and NF-κB signalling in this case.

Here, by applying microarray-based transcriptome analysis, 1,026 genes were found to be differentially expressed (>twofold change) between C6 and C6 TAZ cells. This novel finding has been verified on selected targets using RT-qPCR. The search for responsible mechanisms led to the identification of the transcription factors HOXA5, Pax2, and JUN::JUNB. Transcript levels of these factors themselves were not different between C6 wild-type and C6-TAZ cells. However, Pax2 and also JUN::JUNB appear to be regulated by reversible phosphorylation rather than expression levels (Cai et al., 2002; Meng and Xia, 2011). The effects of tafazzin knock-out on the (phospho)proteome of C6 and other cells should be investigated in further investigations.

By applying Ingenuity Pathway Analysis of transcriptome data Top Diseases and Functions affected by tafazzin knock-out could be identified. Knock-out of tafazzin consistently led to changes in lipid metabolism. The top-regulated Diseases and Functions include [Carbohydrate Metabolism, Lipid Metabolism, Molecular Transport] and [Lipid Metabolism, Small Molecule Biochemistry, Vitamin and Mineral Metabolism]. Of note, Regulation of lipid metabolism/insulin regulation of fatty acid metabolism, Phospholipid metabolism, and Regulation of lipid metabolism/PPAR regulation of lipid metabolism were among the top-regulated pathways that have been identified previously by metabolomics in patients with Barth syndrome (Sandlers et al., 2016). Furthermore, clinical laboratory studies identified a unique biochemical profile specific for Barth patients and which included saturated fatty acids and docosahexaenoic acid (Vernon et al., 2014). All of these data suggest a major change in lipid metabolism in response to lack of tafazzin. Furthermore, in this study, pathways showing highest significance levels include the superpathway of cholesterol biosynthesis and several of its sideways. 14 out of 20 genes of this pathway appeared to be dysregulated in C6-TAZ cells, almost all are down-regulated. In full accordance with a down-regulation of cholesterol biosynthesis, hypocholesterolaemia has been described in some individuals with Barth Syndrome (Vernon et al., 2014). In addition, cholesterol synthesis from palmitate has been shown to depend on CL-synthesis (Hauff et al., 2009). Moreover, plasma concentrations of VLDL-TAGs and LDL-cholesterol concentration were reduced in TAZ knock-out animals (Cole et al., 2016). This points to a compromised hepatic export of cholesterol being present in these patients. C6 TAZ cells contain higher amounts of cholesterol than wild-type C6 cells. It could be speculated that C6 TAZ cells accumulate cholesterol because the efflux is disturbed and/or HDL-based reverse cholesterol transport is not effective under *in vitro* cell culture conditions. This accumulation of cholesterol would partly explain the down-regulation of cholesterol biosynthesis. Alterations in the fatty acid availability in C6 TAZ cells might also contribute to the observed down-regulation of enzymes involved in cholesterol biosynthesis as it has been reported that e.g., oleic acid, linoleic acid inhibit cholesterol biosynthesis (Natali et al., 2007; Priore et al., 2017).

The data presented demonstrate that the full-length rat tafazzin could be functionally distinguished from the Δ5 isoform. Although they both exhibit enzymatic activity and, when re-expressed in tafazzin-knock-out cells, restored the wild-type profile of cardiolipin composition to almost the same extent, it is only the full-length variant that was able to restore the wild-type cells’ proliferation rate. This unexpected finding strongly suggests the existence of non-enzymatic functions of tafazzin. In support of this view, the Δ5 variant showed slightly different behaviour with regard to the differential gene expression that could be induced by tafazzin knock-out. This fact is reflected in the generally weaker (and, therefore, less significant) effects of the Δ5 isoform and in the differences in the regulation of DPP7 and HMGCR when compared to FL-TAZ. Of note, neither re-expression of FL-TAZ or of Δ5-TAZ in tafazzin-deficient C6 cells could reverse all the substantial changes in gene expression observed after TAZ-knock-out. The reason remains to be elucidated fully.

Both isoforms changed the cardiolipin profile from knock-out to normal. It could be speculated that additional mechanisms exist which provoke longer-lasting or persistent changes in gene expression or proliferation. Accumulating evidence links lipid metabolism, and phospholipid metabolism in particular, to regulation of gene expression. Effects could be mediated by epigenetic regulation, by direct effects of lipid-sensing transcription factors (e.g., PGC1A, FXR) or by altered ROS production due to metabolic (mitochondrial) activity (Ferrari et al., 2012; Musille et al., 2013; Silva-Martinez et al., 2016; Chowdhury et al., 2018). Epigenetic regulation might well contribute to incomplete restoration of gene expression despite of the fact that CL-composition is fully restored. This remains, however, to be investigated in future studies. In this context, it seems worth mentioning that HoxA5, a transcription factor the binding sites of which have been found here enriched in up-regulated genes after tafazzin knock-out by means of TF binding site analysis, has been shown to increase DNA methylation in primary adipocytes (Cao et al., 2018).

Supporting the view that indeed epigenetic regulation contributes to differential gene expression observed after knock-out of tafazzin and its long-lasting retention in spite of tafazzin re-expression, the percentage of senescent cells is significantly elevated in C6-TAZ cells, compared to C6 wild-type cells. Recent data demonstrates that cellular senescence is accompanied by epigenetic and chromatin remodelling (Wiley et al., 2016; Nacarelli et al., 2017). Mechanistically, different cellular stressors have been shown to provoke senescence, including oxidative and metabolic stress (Campisi, 2013; Wiley et al., 2016; Nacarelli et al., 2017). How certain individual lipids regulate senescence and how senescence changes the cellular lipidome has been excellently reviewed recently (Hamsanathan and Gurkar, 2022). Oxidized lipids particularly promote the development of cellular senescence (Hamsanathan and Gurkar, 2022). Higher amounts of oxidised CL, used here as a representative marker for lipid oxidation, were detected in C6-TAZ cells. The fact that amounts of oxCL were normalised to a greater extent by re-expressing full length TAZ rather than by re-expressing Δ5 TAZ is in full accordance with the slightly different ability of the isoforms to restore cellular proliferation and gene expression.

## Data Availability

The original contributions presented in the study are publicly available. This data can be found on the Gene Expression Omnibus (GEO) of NCBI with the accession record GSE207004. This includes the raw data from the microarray as well as the processed matrix.
