# Corrigendum: Re-expression of tafazzin isoforms in TAZ-deficient C6 glioma cells restores cardiolipin composition but not proliferation rate and alterations in gene expression

**DOI:** 10.3389/fgene.2022.1009773

**Published:** 2022-08-29

**Authors:** Gayatri Jagirdar, Matthias Elsner, Christian Scharf, Stefan Simm, Katrin Borucki, Daniela Peter, Michael Lalk, Karen Methling, Michael Linnebacher, Mathias Krohn, Carmen Wolke, Uwe Lendeckel

**Affiliations:** ^1^ Institute of Medical Biochemistry and Molecular Biology, University Medicine Greifswald, University of Greifswald, Greifswald, Germany; ^2^ Institute of Clinical Biochemistry, Hannover Medical School, Hannover, Germany; ^3^ Department of Otorhinolaryngology, Head, and Neck Surgery, University Medicine Greifswald, Greifswald, Germany; ^4^ Institute of Bioinformatics, University Medicine Greifswald, Greifswald, Germany; ^5^ Institute of Clinical Chemistry, Department of Pathobiochemistry, Medical Faculty, Otto-von-Guericke University Magdeburg, Magdeburg, Germany; ^6^ Institute of Biochemistry, University of Greifswald, Greifswald, Germany; ^7^ Department of General Surgery, Molecular Oncology, and Immunotherapy, Rostock University Medical Center, Rostock, Germany

**Keywords:** Barth syndrome, cardiolipin, cellular proliferation, gene expression, tafazzin, Barth syndrome (BTHS)

In the published article, there was an error in “Figures 1–5 and Table 1” as published. The figures were not in correct order and some human primers shown in Table 1 were not used in the study. The corrected figures and tables appear below.

**FIGURE 1:**
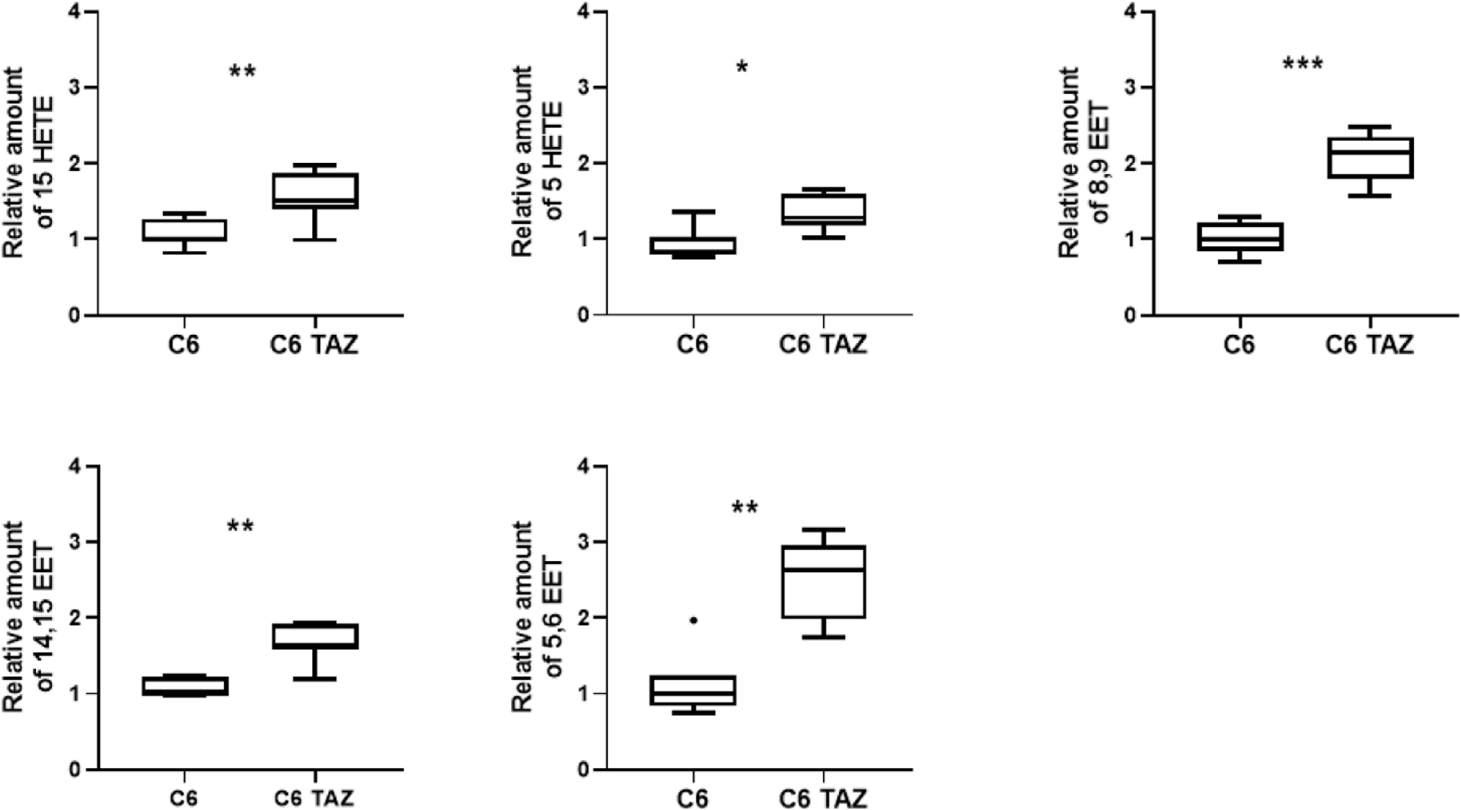
Tafazzin deficiency alters oxylipin levels in C6 cells: Effect of tafazzin knockout on cellular lipids, oxylipins was determined by HPLC-MS/MS. The Data analysis was performed with Agilent Mass Hunter Qualitative and Quantitative Analysis software (version B.08.00 for both). For all detected oxylipins, a relative quantification was done by normalizing the area of the metabolite signal to the area of the signal of the internal standard compound (relative amount). Data is represented as median,quartile and interquartile range for *n* = 4.

**FIGURE 2:**
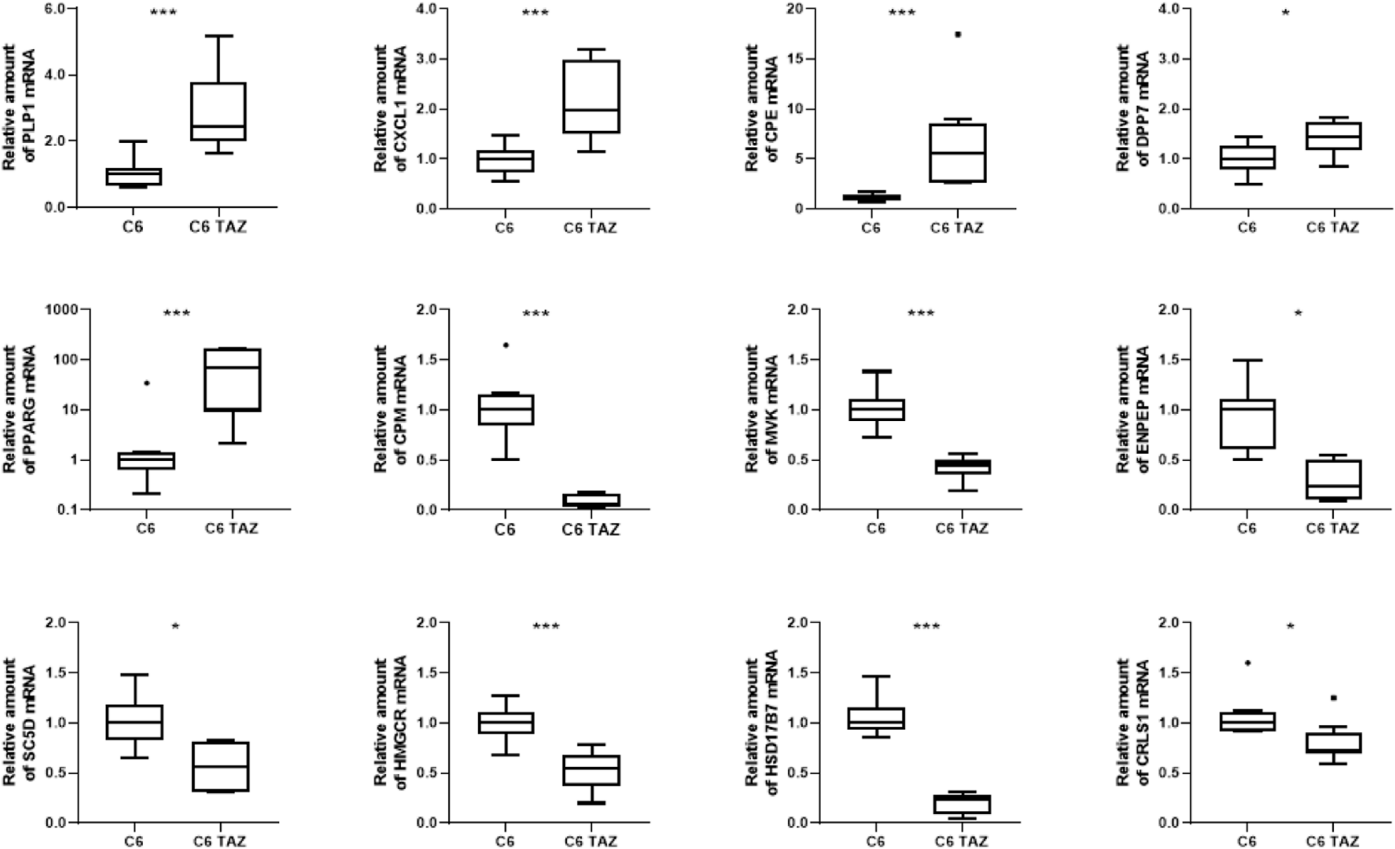
Tafazzin deficiency alters gene expression in C6 cells: Quantification levels of indicated genes in C6 and C6 TAZ cells was preformed and the amount of mRNA was determined by using RT-qPCR and normalized to RPL13a. Data is given as boxplot with median, quartile, and interquartile range (*p < 0.05; **p < 0.01; ***p < 0.001). Outliers are indicated using Tukey method. *n* = 8.

**FIGURE 3:**
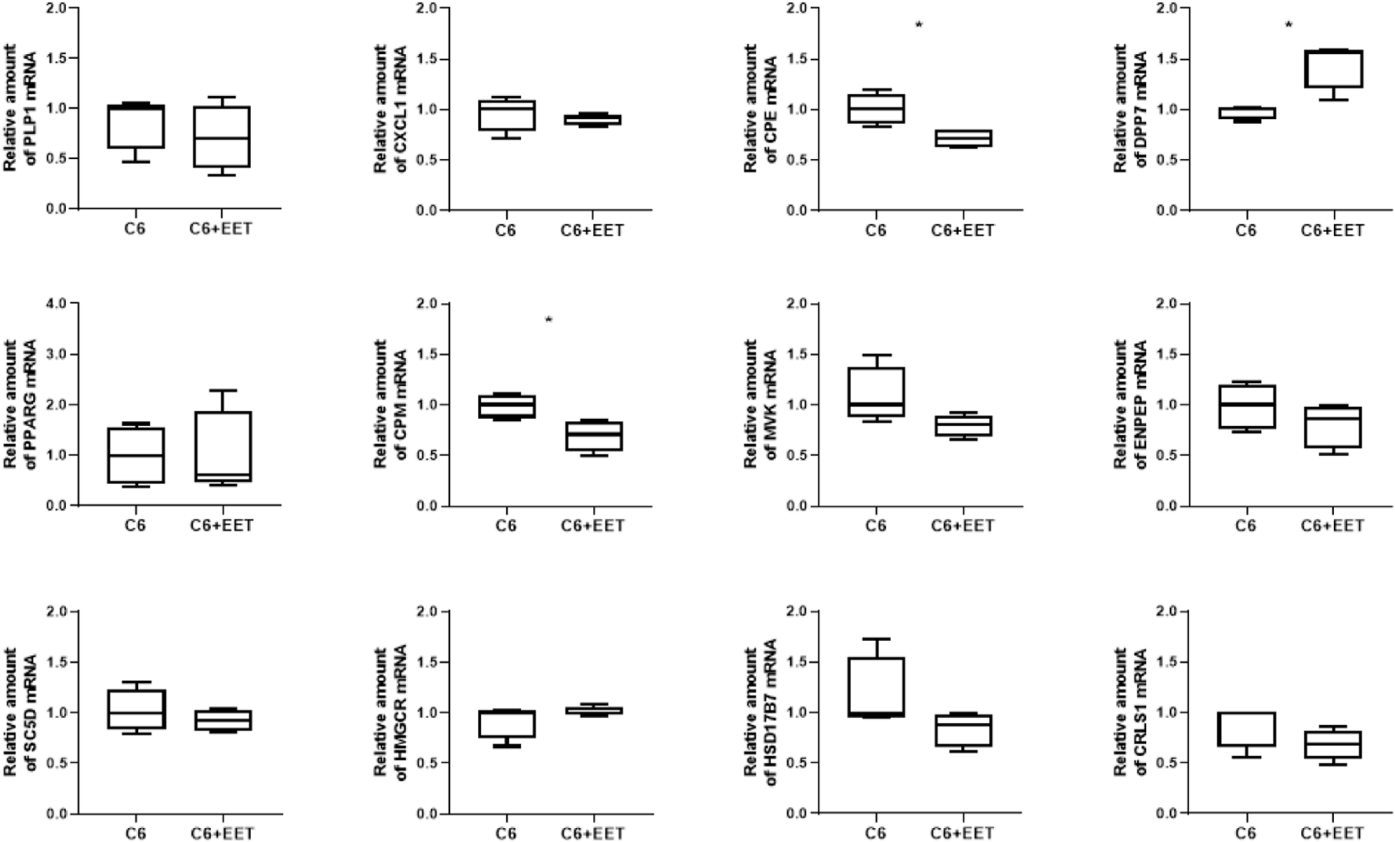
Oxylipins regulates TAZ dependent gene expression in C6 cells: Relative gene expression of C6 cells treated with ethanol (control) and C6 cells with 3 μM EET incubated at 37°C for 24 h. The amount of Mrna is determined by qpcr that is normalized to RPL13a. Data is represented as boxplot with median, quartile and interquartile range. Asterisks represent significant change.

**FIGURE 4:**
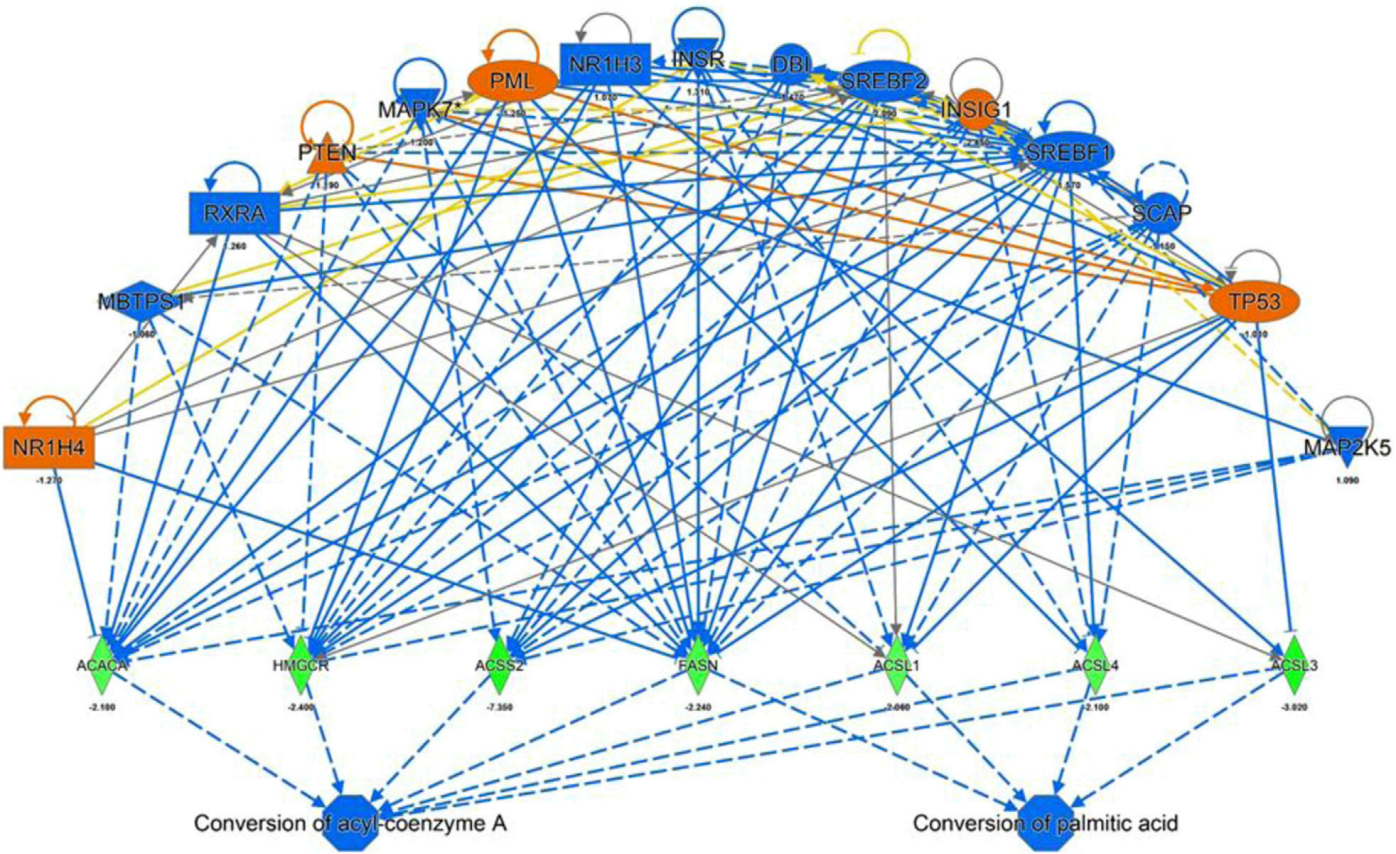
IPA Upstream Regulators Analysis for identification of upstream regulators that may be responsible for changes in gene expression observed for ACACA, HMGCR, ACSS2, FASN, ACSL 1,3 and 4 (predicted activation (orange) predicted inhibition (blue) of the regulators leads to a decreased expression (green)).

**FIGURE 5:**
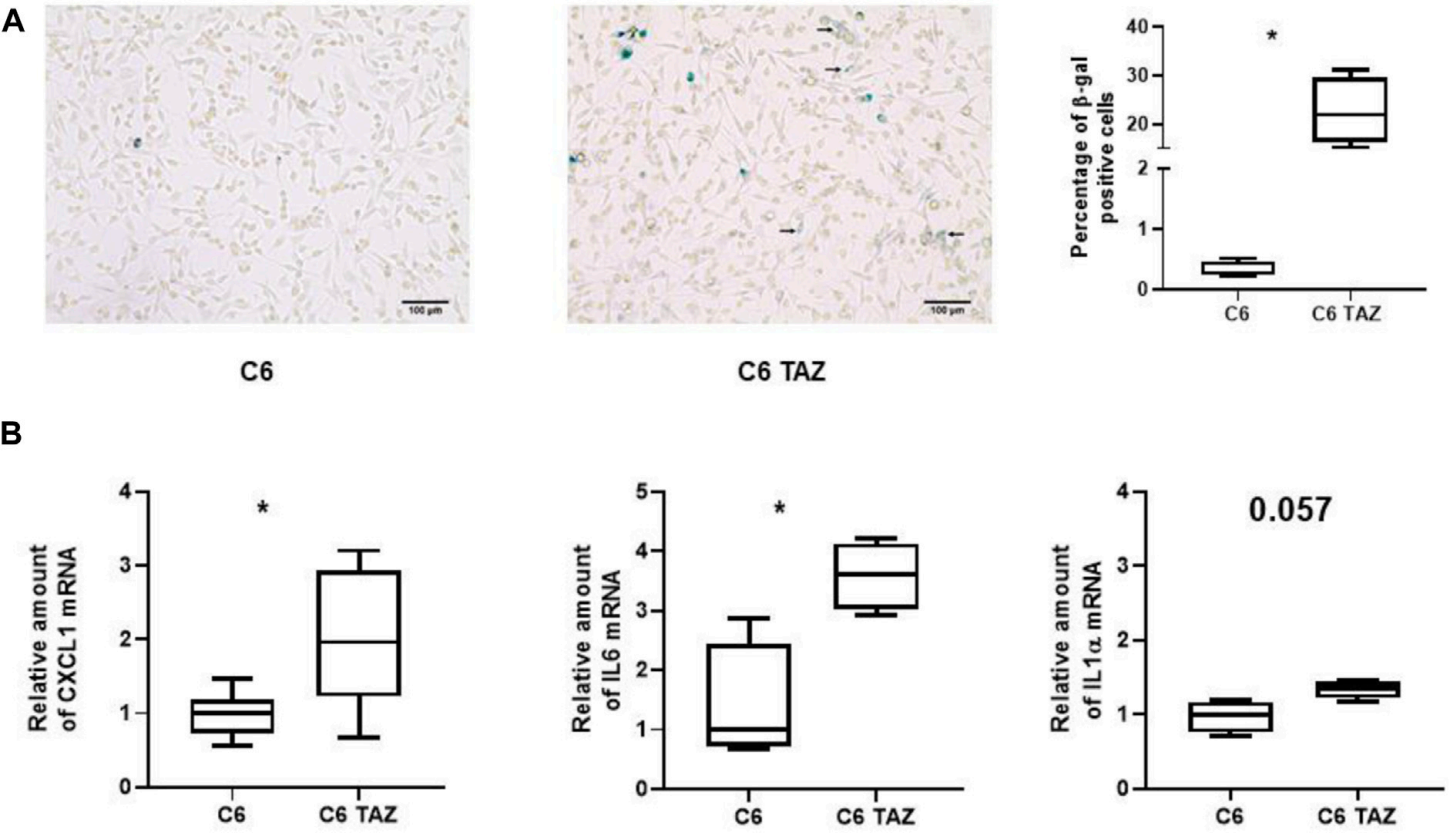
**(A)** Left: Microscopic images of β-gal positive cells (blue color) in C6 and C6 TAZ cells. Right: Percentage of β-gal positive cells is given as boxplot (* indicates *p* < 0.05). **(B)** Upregulation of SASP genes: Amounts of mrnas of CXCL1, IL6, and IL1α are elevated in C6 TAZ cells (mrna expression normalized to RPL13a; *n* = 4, *p* = 0.029).

**TABLE 1:** List of primers used in the study.

Gene	Upstream	Downstream
rPLP1	GCC CTG ACT GTT GTA TGG CT	AGG GAA ACT AGT GTG GCT GC
rCXCL1	CTG CAC CCA AAC CGA AGT CA	GAC GCC ATC GGT GCA ATC TA
rCPE	ACC TCC CTG TCG CAA GAA TG	CCA TCC TTA GCC GAG GTG AC
rDPP7	GGG GAG CAC ATC ACC TAG AC	GAA GGC TGC TAC TTA GGC CC
rPPARG	TCA AAG TAG AGC CTG CGT CC	TGG CAT TGT GAG ACA TCC CC
rCPM	CGA GGC AAG ATT GAC CCA GT	CAG CTC GTT TCC TTT CAC GC
rMVK	TCA TGG TGT GGT CGG AAC TG	GGT ACT TCG TGG GAC CTT GG
rENPEP	CCT CAC ATC CGG TGG TTG TC	TGG GTG ACG TTC TGC TTT CC
rSC5D	GAC CCT GGC AGC ACT GTA AT	GGT CGG CTT TCC TGG CTA AT
rHMGCR	TAG AGA TCG GAA CCG TGG GT	GCC CGT GTT TCA GTC CAG TA
rHSD17B7	CTG ACC AAA TAC TTG AGC GGC	TAG GAG GAG AGA TCA TCA TGG C
rCRLS1	GCA TTC ACT ACA GCT GCG TC	GCT GAA CAC CAA GAT CGG GA
rIL6	TCA TTC TGT CTC GAG CCC AC	GTC TCC TCT CCG GAC TTG TG
rILa	AGT GGA ACC AGC CCG ACA TA	TAT CCT ACC CAT CCG GCA CT
RPLP13a	CTG GTA CTT CCA CCC GAC CTC	GGA TCC CTC CAC CCT ATG ACA

