# Supplementary material for: Re-Expression of Tafazzin Isoforms in TAZ-Deficient C6 Glioma Cells Restores Cardiolipin Composition but Not Proliferation Rate and Alterations in Gene Expression
Source: Front Genet. 2022 Jul 25;13:931017. doi: 10.3389/fgene.2022.931017 (PMC9358009; doi:10.3389/fgene.2022.931017)
Supplement: Supplementary file 1 [file DataSheet2.pdf]

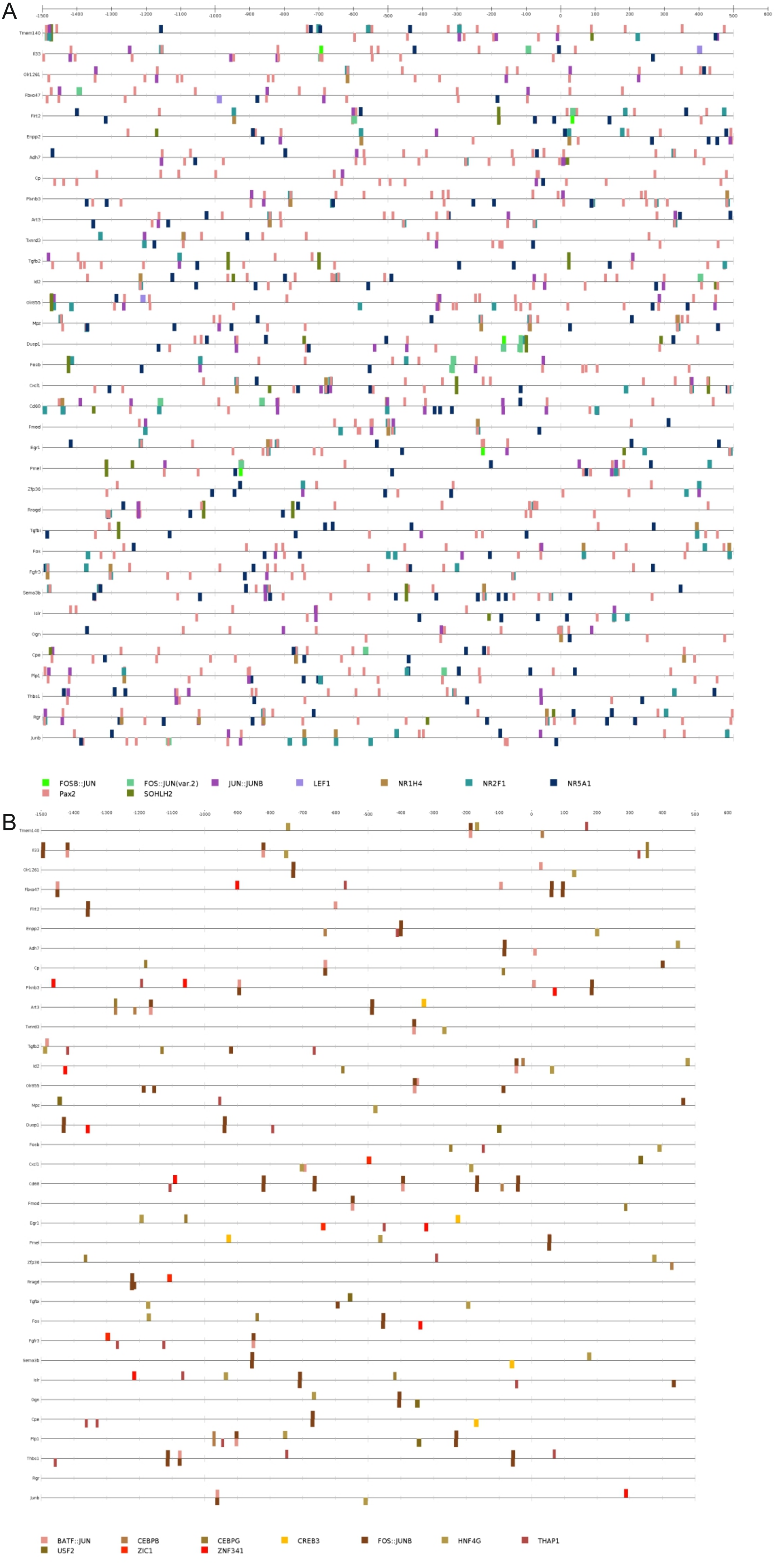
A
-1500
-1400
-1300
-1200
-1100
-1000
-900
-800
-700
-600
-500
-400
-300
-200
-100
0
100
200
300
400
500
600
Tmem140
Il33
Olr1261
Fbxo47
Flrt2
Enpp2
Adh7
Cp
Plxnb3
Art3
Txnrd3
Tgfb2
Id2
Olr855
Mpz
Dusp1
Fosb
Cxcl1
Cd68
Fmod
Egr1
Pmel
Zfp36
Rragd
Tgfbi
Fos
Fgfr3
Sema3b
Islr
Ogn
Cpe
Plp1
Thbs1
Rgr
Junb
FOSB::JUN
FOS::JUN(var.2)
JUN::JUNB
LEF1
NR1H4
NR2F1
NR5A1
Pax2
SOHLH2
B
BATF::JUN
CEBPB
CEBPG
CREB3
FOS::JUNB
HNF4G
THAP1
USF2
ZIC1
ZNF341

Figure S2: Shown are **A)** the 4fold upregulated gene sequences of 1,500 nt upstream and 500 nt downstream and the occurence of most enriched TF binding sites and **B)** unspecific enriched TF binding profiles in the upregulated gene lists using the background genelist (foldchange between -1 and +1).
