# Supplementary material for: Re-Expression of Tafazzin Isoforms in TAZ-Deficient C6 Glioma Cells Restores Cardiolipin Composition but Not Proliferation Rate and Alterations in Gene Expression
Source: Front Genet. 2022 Jul 25;13:931017. doi: 10.3389/fgene.2022.931017 (PMC9358009; doi:10.3389/fgene.2022.931017)
Supplement: Supplementary file 2 [file DataSheet4.pdf]

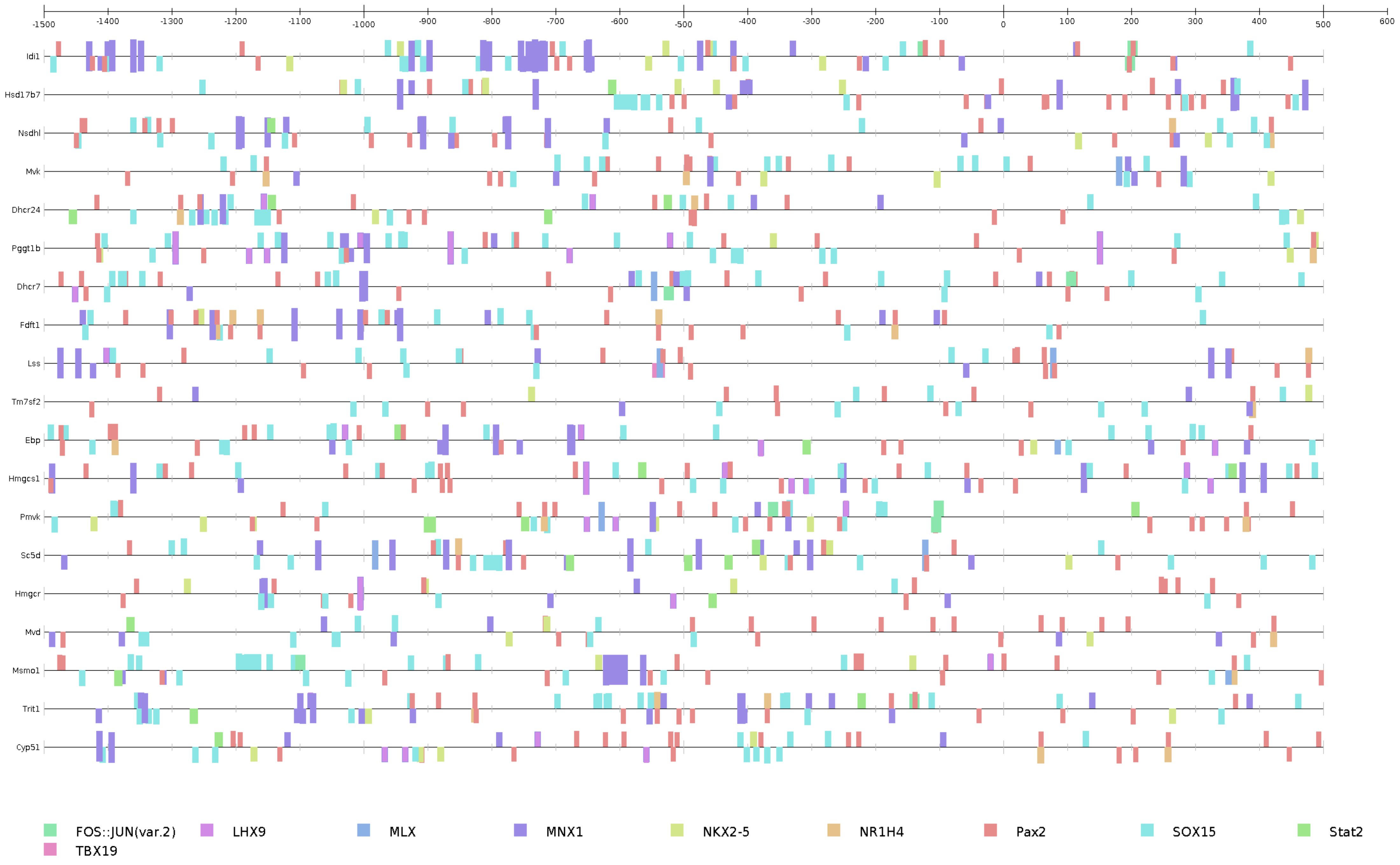

-1500
-1400
-1300
-1200
-1100
-1000
-900
-800
-700
-600
-500
-400
-300
-200
-100
0
100
200
300
400
500
600
Idi1
Hsd17b7
Nsdhl
Mvk
Dhcr24
Pggt1b
Dhcr7
Fdft1
Lss
Tm7sf2
Ebp
Hmgcs1
Pmvk
Sc5d
Hmgcr
Mvd
Msmo1
Trit1
Cyp51
FOS::JUN(var.2)
LHX9
MLX
MNX1
NKX2-5
NR1H4
Pax2
SOX15
Stat2
TBX19

Figure S4: The related sequences to the Cholesterol pathway and their 1,500 nt upstream and 500 nt downstream sequences are visualized with the position and occurence of TF binding sites (Top enriched TFs represented in different colors).
