# Supplementary material for: Re-Expression of Tafazzin Isoforms in TAZ-Deficient C6 Glioma Cells Restores Cardiolipin Composition but Not Proliferation Rate and Alterations in Gene Expression
Source: Front Genet. 2022 Jul 25;13:931017. doi: 10.3389/fgene.2022.931017 (PMC9358009; doi:10.3389/fgene.2022.931017)
Supplement: Supplementary file 3 [file DataSheet3.pdf]

Superpathway of Cholesterol Biosynthesis

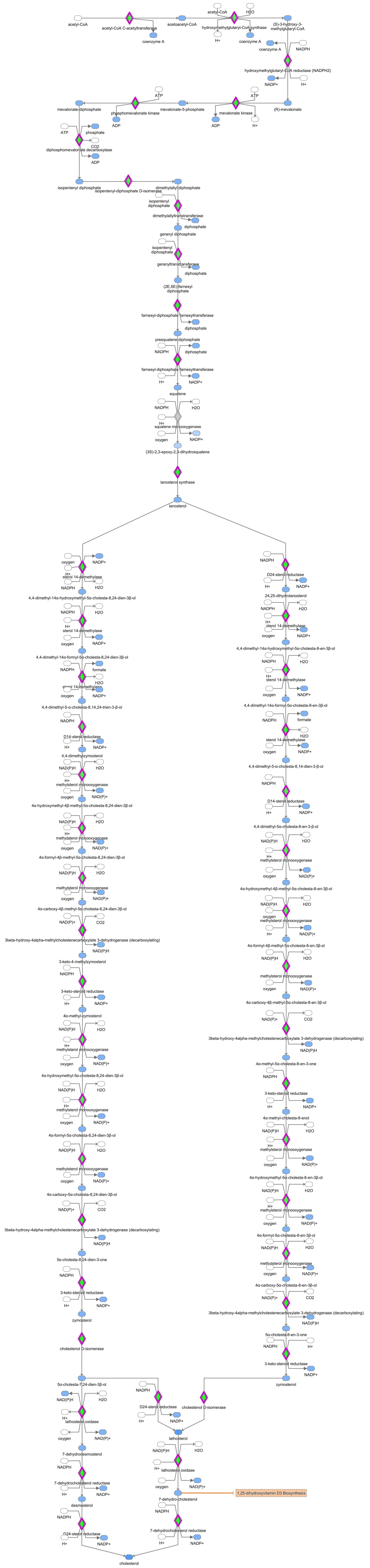

Adapted from HumanCyc © 2000-2021 QIAGEN. All rights reserved.

Figure S3: Super-pathway of Cholesterol biosynthesis
