# Supplementary material for: Re-Expression of Tafazzin Isoforms in TAZ-Deficient C6 Glioma Cells Restores Cardiolipin Composition but Not Proliferation Rate and Alterations in Gene Expression
Source: Front Genet. 2022 Jul 25;13:931017. doi: 10.3389/fgene.2022.931017 (PMC9358009; doi:10.3389/fgene.2022.931017)
Supplement: Supplementary file 4 [file DataSheet1.PDF]

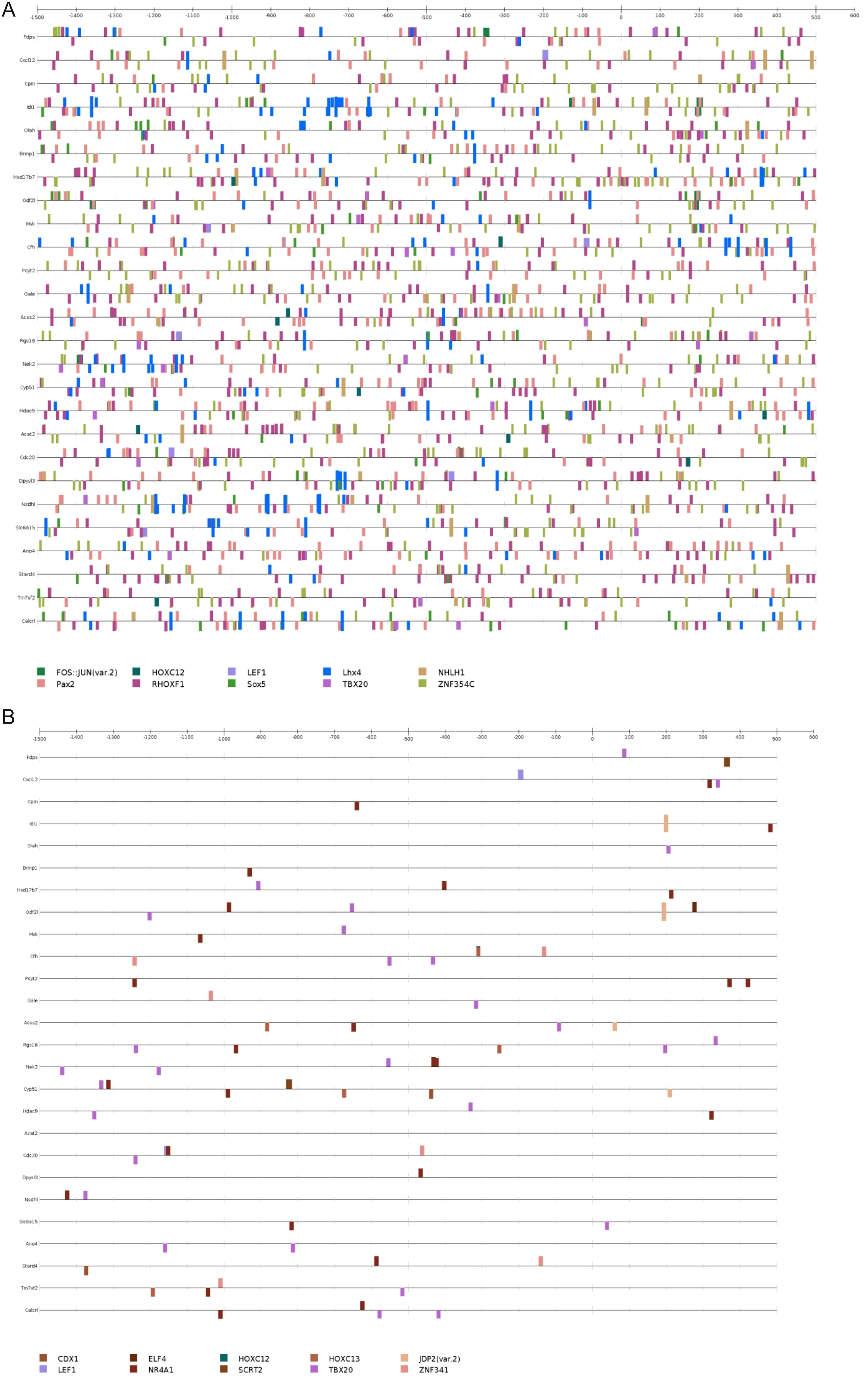
A
-1500 -1400 -1300 -1200 -1100 -1000 -900 -800 -700 -600 -500 -400 -300 -200 -100 0 100 200 300 400 500 600
Fdps
Cxcl12
Cpm
Idi1
Olah
Brinp1
Hsd17b7
Odf2l
Mvk
Cfh
Pcyt2
Gale
Acss2
Rgs16
Nek2
Cyp51
Hdac9
Acat2
Cdc20
Dpysl3
Nsdhl
Slc6a15
Ano4
Stard4
Tm7sf2
Calcrl
FOS::JUN(var.2)
HOXC12
LEF1
Lhx4
NHLH1
Pax2
RHOXF1
Sox5
TBX20
ZNF354C
B
-1500 -1400 -1300 -1200 -1100 -1000 -900 -800 -700 -600 -500 -400 -300 -200 -100 0 100 200 300 400 500 600
Fdps
Cxcl12
Cpm
Idi1
Olah
Brinp1
Hsd17b7
Odf2l
Mvk
Cfh
Pcyt2
Gale
Acss2
Rgs16
Nek2
Cyp51
Hdac9
Acat2
Cdc20
Dpysl3
Nsdhl
Slc6a15
Ano4
Stard4
Tm7sf2
Calcrl
CDX1
ELF4
HOXC12
HOXC13
JDP2(var.2)
LEF1
NR4A1
SCRT2
TBX20
ZNF341

Figure S1: Represented are the TF binding sites within the 1,500 nt upstream and 500 nt downstream sequences of 4 fold downregulated genes from the microarray expression analysis. **A)** The 4fold downregulated genes and the occurence of most enriched TF binding sites are shown. **B)** Unspecific TF binding profiles in the downregulated gene lists were identified using a background genelist consisting of the non-affected genes in the microarray expression analysis (foldchange between -1 and +1).
